# EIF4A3-mediated biogenesis of *circSTX6* promotes bladder cancer metastasis and cisplatin resistance

**DOI:** 10.1186/s13046-023-02932-6

**Published:** 2024-01-02

**Authors:** Wenjie Wei, Kan Liu, Xing Huang, Shuo Tian, Hanfeng Wang, Chi Zhang, Jiali Ye, Yuhao Dong, Ziyan An, Xin Ma, Baojun Wang, Yan Huang, Xu Zhang

**Affiliations:** 1 Department of Urology, The Third Medical Center, Chinese PLA General Hospital, Beijing, 100039 China; 2 Medical School of PLA, Beijing, 100853 China

**Keywords:** Bladder cancer, *circSTX6*, *miR-515-3p*, PABPC1, SUZ12, CDDP

## Abstract

**Background:**

Cisplatin (CDDP)-based chemotherapy is a standard first-line treatment for metastatic bladder cancer (BCa) patients, and chemoresistance remains a major challenge in clinical practice. Circular RNAs (circRNAs) have emerged as essential regulators in carcinogenesis and cancer progression. However, the role of circRNAs in mediating CDDP chemosensitivity has yet to be well elucidated in BCa.

**Methods:**

*CircSTX6* (*hsa_circ_0007905*) was identified by mining the public circRNA datasets and verified by Sanger sequencing, agarose gel electrophoresis, RNase R treatment and qRT-PCR assays. Then, function experiments were performed to evaluate the effects of *circSTX6* on BCa metastasis. Luciferase reporter assay, RNA pull-down, RNA immunoprecipitation (RIP), RNA stability assay, Fluorescence in situ hybridization (FISH) and Immunofluorescence (IF) were conducted to evaluate the interaction among *circSTX6*, *miR-515-3p*, PABPC1 and *SUZ12*. Animal experiments were performed to explore the function of *circSTX6* in tumor metastasis and CDDP sensitivity.

**Results:**

We identified that *circSTX6* was significantly upregulated in clinical samples and cells of BCa. Functionally, *circSTX6* promoted cell migration and invasion both in vitro and in vivo*.* Mechanistically, *circSTX6* could act as a *miR-515-3p* sponge and abolish its effect on *SUZ12*. Moreover, *circSTX6* was confirmed to increase the stability of *SUZ12* mRNA by interacting with a mRNA stabilizer PABPC1 and subsequently promote the expression of SUZ12. Importantly, silencing of *circSTX6* improved the chemosensitivity of CDDP-resistant bladder cancer cells to CDDP. Furthermore, in vivo analysis supported that knockdown of *circSTX6* attenuated CDDP resistance in BCa tumors.

**Conclusion:**

These studies demonstrate that *circSTX6* plays a pivotal role in BCa metastasis and chemoresistance, and has potential to serve as a therapeutic target for treatment of BCa.

**Supplementary Information:**

The online version contains supplementary material available at 10.1186/s13046-023-02932-6.

## Background

Bladder cancer is overall the ninth most common malignancy and the most prevalent cancer in urinary system, with an estimated 81,180 new cases and 17,100 deaths in 2022 in the United States [1, 2]. Approximately 25% of new patients are diagnosed as muscle-invasive or metastatic disease [3]. The current gold standard for the diagnosis of BCa involves the use of cystoscopy and cytology. Recently, a new non-invasive urine tumor DNA methylation detection could improve the diagnostic rate of early tumors [4]. Transurethral resection of bladder tumors and radical cystectomy are recommended for early-stage patients, but when the tumors deteriorated to advanced stages, the cancers are no longer curable by surgical treatments [5]. Clinically, cisplatin (CDDP)-based chemotherapy has become the standard frontline treatment for patients with advanced BCa. However, patients always obtain drug resistance after several cycles of CDDP-based treatment, which limiting overall clinical efficacy [6, 7]. Under these circumstances, a better comprehension of the molecular mediators and regulatory mechanisms underlying CDDP resistance is of great clinical importance for BCa patients.

Circular RNAs (circRNAs), a category of noncoding RNAs, are derived from back-splicing of precursor mRNAs, with a covalently closed loop structures without 5’caps and 3’polyadenylated tails [8, 9]. In recent years, large amounts of circRNAs have been successfully discovered and identified in a tissue-specific or cell type-specific manner by high-throughput sequencing analysis [10, 11]. Accumulating evidence indicates that circRNAs play important roles in multiple biological functions, such as cell proliferation, migration, invasion and chemosensitivity [12–14]. Our previous studies demonstrate that some dysregulated circRNAs play a vital role in chemotherapy sensitivity of BCa [15, 16]. It has been reported that circRNAs may regulate gene expression by sponging miRNAs in a complementary base-pairing manner [17]. In addition, circRNAs can also interact with RNA-binding proteins (RBPs) to form specific complexes that subsequently mediate the function of associated proteins [18]. Increasing evidence indicates that circRNAs could also exert their functions through regulating transcription and encoding peptide [19, 20]. Notably, recent studies have depicted an emerging expression profile of circRNAs in BCa, with tumor-promoting or tumor-inhibiting properties [21, 22]. However, the functions and regulatory mechanisms of circRNAs in modulating CDDP resistance in BCa still remain largely enigmatic and need to be further explored.

In the present study, we identified a circRNA derived from the *STX6* gene, *hsa_circ_0007905* designated as *circSTX6*, by analyzing expression profiles of circRNAs in BCa. *CircSTX6* was frequently upregulated in BCa tissues compared with paired noncancerous adjacent tissues, and increase of eukaryotic initiation factor 4A3 (EIF4A3) contributed to the upregulation of *circSTX6*. Subsequent studies showed that *circSTX6* could directly sponge *miR-515-3p* to upregulate SUZ12 expression and consequently promote the metastasis of BCa in vitro and in vivo. Importantly, *circSTX6* increased the stability of *SUZ12* mRNA by strengthening its interaction with poly(A) binding protein cytoplasmic 1 (PABPC1), leading to enhanced expression of SUZ12. Notably, knockdown of *circSTX6* could promote the sensitivity of BCa to CDDP chemotherapy in vitro and in vivo. Taken together, our study delineated an important role of *circSTX6* in tumor progression and CDDP resistance, providing a potential therapeutic target for overcoming metastasis and chemoresistance in BCa.

## Methods

### Cell culture and treatment

Human normal bladder epithelial cell line SV-HUC-1 was purchased from ATCC. The human urinary bladder transitional cell carcinoma cell lines T24, RT4, UMUC3 and 5637 were purchased from ATCC. EJ cells were obtained from the Institute of Biochemistry and Cell Biology of Chinese Academy of Sciences (Shanghai, China). CDDP-resistant cell lines (EJ-CDDP) were derived from our previous studies [15]. All cells were maintained in a humidified incubator with 5% CO_2_ at 37℃. The SV-HUC-1 cells were cultured in F12K medium (Procell, China). The T24, EJ, RT4, 5637 and EJ-CDDP cells were cultured in RPMI 1640 medium (Procell, China). The UMUC3 cells were cultured in DMEM (Procell, China). All medium was supplemented with 10% FBS (Vazyme, China) and 1% penicillin/streptomycin (Procell, China). All cell lines were confirmed negative for *Mycoplasma* contamination. Cisplatin (CDDP) and actinomycin D (ActD) were purchased from Sigma-Aldrich (USA) and solubilized in DMSO.

### Clinical samples

A total of 16 pairs of bladder cancer tissues and NATs were obtained from patients who had undergone surgical resection at The Department of Urology of the Chinese PLA General Hospital (Beijing, China). The histologic and pathologic type of all tissues were confirmed and classified by three experienced clinical pathologists independently. The samples were obtained with written informed consent of all patients before the research started and were approved by the Institutional review board of the Chinese PLA General Hospital. All samples were immediately snap-frozen in liquid nitrogen and stored at -80 °C. The characteristics of BCa patients were listed in Supplementary Table 1.

### RNA preparation, RNase R, and qRT-PCR

Total RNA was isolated using FastPure Cell/Tissue Total RNA Isolation Kit V2 (Vazyme, China) according to the manufacturer’s instructions from either clinical samples or cultured cells. Concentration and purity for each RNA samples were detected by 260/280 ratio on the ND100-C instrument (MIULAB, China). RNase R treatment was performed for 15 min at 37 °C using RNase R (Epicentre, USA) 3 U/µg. RNA was reverse-transcribed using HiScript III RT SuperMix for qPCR (Vazyme, China). ChamQ Universal SYBR qPCR Master Mix (Vazyme, China) was used for qRT-PCR. The primers are listed in Supplementary Table 2. The circRNA and mRNA levels were normalized by GAPDH.

### Plasmids and transfection

To construct *circSTX6* overexpression plasmids, human *circSTX6* cDNA was synthesized and cloned into pcDNA3.1(+) CircRNA Mini Vector (addgene, USA). The pcDNA3.1(+) CircRNA Mini Vector contained a front circular frame and a back circular frame. Short hairpin RNAs targeting circSTX6, SUZ12 and PABPC1 were synthesized by BIOMED, and were cloned into the pLKO.1 vector (addgene, USA). The human *SUZ12* and *PABPC1* cDNA were synthesized by BIOMED, which were cloned into pLVX-IRES-ZsGreen1 vector (Beijing qualityard biotechnology, China) to construct overexpression plasmid. The plasmids for overexpression and knockdown of EIF4A3 were constructed in our previous studies [15]. The package of virus and transfection of plasmid were performed using jetPRIME Transfection Reagent (Polyplus, France) according to the manufacturer’s instructions. Stable transfection was established and maintained by culturing BCa cells in regular medium supplemented with puromycin or neomycin. The sequences of shRNA were listed in Supplementary Table 2.

## RNA pulldown assay

Pull-down assay was performed as described in our previously studies [15]. In brief, about 10^7^ cells were washed in ice-cold PBS, lysed in 500 µl Co-IP buffer (Thermo Scientific, USA) supplemented with a cocktail, PMSF, and RNase inhibitor (Solarbio, China), and then incubated with 3 µg biotinylated DNA *circSTX6* sense probe or antisense probe for 2 h at room temperature. Then the reactions were mixed with 50 µl Streptavidin C1 magnetic beads (Invitrogen, USA) for another hour, which were prewashed twice with tris buffer. The beads were washed briefly with Co-IP buffer for five times. Finally, the retrieved RNA was used for qRT-PCR analysis. Biotinylated *circSTX6* sense probes or antisense probes (Supplementary Table 2) were synthesized by RiboBio (Guangzhou, China). The RNA-protein binding mixture was boiled in SDS buffer and the released proteins were detected by western blot and silver staining. Mass spectrometry was conducted at Shanghai Luming biological technology co.,LTD (Supplementary Table 3).

### Biotin-labeled miRNA capture

Stably overexpressed *circSTX6* BCa cells were transfected with biotinylated wild-type or mutant miR-515-3p using Lipofectamine RNAiMax. After 48 h of transfection, the cells were harvested, sonicated and incubated with Streptavidin C1 magnetic beads (Invitrogen, USA) at 4 °C on the rotator overnight. The bound RNAs were purified using the FastPure Cell/Tissue Total RNA Isolation Kit V2 (Vazyme, China) and the abundance of *circSTX6* in bound fractions was tested by qRT-PCR.

### Western blot analysis

Protein extracts were isolated from BCa cells with RIPA lysis buffer containing protease inhibitor cocktail and PMSF. An equal amount of total protein lysates was separated by SDS-PAGE gel and transferred onto PVDF membranes. The membranes were incubated with primary antibodies against Actin (BE0021, 1:5,000, bioeasytech), EIF4A3 (catalog no. 17504-1-AP, 1:1,000, Proteintech), SUZ12 (catalog no. 20366-1-AP, 1:1,000, Proteintech), PABPC1 (catalog no. 10970-1-AP, 1:1,500, Proteintech) overnight at 4 °C, followed by an incubation with HRP-conjugated secondary goat anti-mouse (catalog no. SA00001-1, 1:4,000, Proteintech) or goat anti-rabbit (catalog no. SA00001-2, 1:4,000, Proteintech) for 1 h at room temperature. Finally, the protein bands were visualized using ECL substrate kit via QuickChemi 5200 Imaging System.

### RNA immunoprecipitation

Briefly, the proteinA/G magnetic beads (MCE, USA) were incubated with anti-EIF4A3 antibodies (Proteintech, USA), anti-PABPC1 antibodies (Proteintech, USA) or IgG negative control antibody (Proteintech, USA). Subsequently, BCa cells were lysed and incubated with the corresponding antibody-coated beads. Co-precipitated RNAs were then purified and measured by qRT-PCR for enrichment. The primer sequences were listed in Supplementary Table 2.

### Wound healing, migration and invasion assays

For wound healing assay, straight scratch was made with a sterile 200 µL pipette tips (0 h) in the six-well plates and photographed immediately, and then the cells were cultured with serum-free medium. After 24 h, images of wounds were captured, and the distance was measured and normalized to the 0 h control as the relative migration rate.

For transwell migration and invasion assays, a 24-well transwell chamber (Costar, USA) with or without Matrigel (BD Science, USA) was used to detect cell invasive and migratory abilities, respectively. Cells were suspended in 200 µL serum-free medium and added to the upper chambers (5 × 10^4^ cells per well for migration, and 1 × 10^5^ per well for invasion). About 600 µL of medium supplemented with 10% FBS was applied to the lower chambers. After incubation for 24 or 48 h, cells in the upper chamber were softly removed with cotton swabs, and cells that migrated to the lower membrane surface were fixed with 4% paraformaldehyde and stained with crystal violet in PBS for photographing and counting. The migrated and invaded cells were counted in three randomly selected fields.

### Sphere-formation assay

Self-renewal of cancer stem cells was assessed by tumor sphere formation. Briefly, cells were plated in ultra-low adhesion 12-well plates at a density of 1000 cells/well and grown in a serum-free DMEM/F12 medium (Procell, China) containing 20 ng/ml of epidermal growth factor, 5 µg/ml of insulin, 0.4% bovine serum albumin, 2% B27 and 1% penicillin/streptomycin. After 10 days in culture, the number of spheres was quantified by counting spheres under a phase contrast microscope (Olympus, Japan).

### EdU assay

EdU assay was performed using Cell-Light™ EdU ApolloR567 In Vitro Kit (RiboBio, China) according to manufacturer’s protocols. Briefly, BCa cells were seeded in 96-well plate and labeled with 50 µM EdU for an additional 2 h. Then, the cells were fixed with 4% paraformaldehyde in PBS and stained. Finally, the cells were subjected to nuclear staining with DAPI for 15 min at RT. The images were captured with an Olympus FSX100 microscope (Olympus, Japan) and cell proliferation rate was calculated.

### Cell viability analysis

The BCa cells were seeded in 96-well plate with 5000 cells per well, and were cultured at 37˚C overnight. Then, the cells were treated with a series of dilute concentrations of cisplatin for 24 h. To evaluate the cytotoxicity of cisplatin, cell viability was tested by CCK-8 kit (Dojindo, Japan) according to the manufacturer’s instructions.

### RNA sequence and bioinformatics analysis

The EJ and UMUC3 cells stably transfected with circSTX6 shRNA#1 and corresponding control were harvested, and then we extracted their total RNA. A high-quality RNA sample was used to construct a sequencing library in Majorbio Technologies (Shanghai, China). The results of RNA-seq were listed in Supplementary Table 4. The databases of CircInteractome and Circbank were used to predict the potential miRNAs bound with circSTX6. The potential target genes mediated by miR-515-3p were acquired from TargetScan, miRDB and miRTarBase. In addition, RBPDB and RBPmap were used to predict the RBPs interacted with circSTX6.

### Fluorescence in situ hybridization (FISH) and immunofluorescence (IF)

Cy3-labeled probes targeting *circSTX6* and FAM-labeled *miR-515-3p* probes were designed and synthesized by RiboBio Technology Co. Ltd. The signals of the probes were detected by the FISH Kit (RiboBio, China) according to the manufacturer’s instructions. Briefly, BCa cells were fixed with 4% paraformaldehyde and permeabilized with 0.5% Triton X-100. After prehybridization, cells were hybridized at 37 °C overnight in a dark, humid chamber. Nuclei were counterstained with DAPI. For IF, cells were fixed, permeabilized, blocked with 5% BSA for 30 min at 37 °C, and then incubated with PABPC1 (catalog no. 10970-1-AP, 1:50, Proteintech) or γH2AX (#9718, 1:200, CST) primary antibody overnight at 4 °C. After washing twice with PBS and then cells were incubated with the Alexa Fluor 488 or the Alexa Fluor 594 (ABclonal, China) for 1 h at 37 °C, followed sealing with parafilm containing DAPI. Fluorescent images were acquired using a confocal microscope (Olympus, China).

### Dual-luciferase reporter assay

BCa Cells were seeded into 24-well plates (5 × 10^4^ cells per well). The cells were co-transfected with psiCHECK2 *circSTX6* 3’-UTR-wide type/mutant reporter vector and *miR-515-3p* to examine the miRNA binding abilities. The psiCHECK2 *SUZ12* 3’-UTR-wide type/mutant reporter vector was co-transfected with *miR-515-3p* to determine the 3’-UTR activity of *SUZ12*. On the other hand, the psiCHECK2 *SUZ12* 3’-UTR reporter vector was co-transfected with PABPC1 overexpression plasmid/shRNA plasmids or circSTX6 overexpression plasmid to determine the 3’-UTR activity of *SUZ12*. After transient transfection for 48 h, the firefly and Renilla luciferase activities were measured with a dual-luciferase reporter assay system (Promega, USA) according to the manufacturer′s protocol. Renilla luciferase activity was normalized to the luminescence of firefly luciferase.

### RNA stability assay

RNA stability was determined using ActD treatment. In short, cells were seeded in 6-well plates. Up to 60% confluency after 24 h, cells were treated with 5 µg/ml of ActD for 0, 3, 6, 9 or 12 h before RNA isolation. The *SUZ12* mRNA levels were detected by qRT-PCR assay. The half-life of *SUZ12* mRNA was estimated according to our previously published paper [16].

### Animal experiments

All mice used in this study were housed in obedience with the institutional guidelines and approved by the Institutional Animal Care and Use Committee of the Chinese PLA General Hospital. For the in vivo tumor metastasis studies, six-week-old male BALB/c nude mice were preserved in SPF-grade animal laboratory and randomly divided into three groups. EJ cells that stably transfected with *circSTX6* knockdown plasmid or scramble plasmid were injected into the nude mice via tail vein (2 × 10^6^ cells per mouse). After several weeks, all mice were sacrificed. The In-Vivo FX PRO (Bruker, USA) was used to obtain fluorescence images of xenografts in nude mice.

For in vivo drug studies, BALB/c nude mice (6 weeks old) were randomly divided into four groups, and stably downregulated *circSTX6* or control EJ cells (2 × 10^6^ cells per mouse) were injected subcutaneously into the right flank of the nude mice. For drug treatment, mice were treated with PBS or CDDP via intraperitoneal injection three-times weekly at the dose of 1 mg/kg. Tumor volume was measured with calipers and calculated as length × width^2^ × 0.5. After 4 weeks, all mice were sacrificed, and the tumors were excised and weighed.

### Statistical analysis

Statistical analysis was performed using GraphPad Prism 8.0. Data were shown as the mean ± standard deviation (SD). Kaplan-Meier method and log-rank test were used to calculate overall survival rates. The significance of intergroup differences was determined with Student’s t-test or one-way ANOVA. Correlations were analyzed by Pearson’s correlation test. *P* < 0.05 was considered statistically significant and was marked with an asterisk.

## Results

### Expression and characterization of *circSTX6* in BCa cells and tissues

**Fig. 1:**
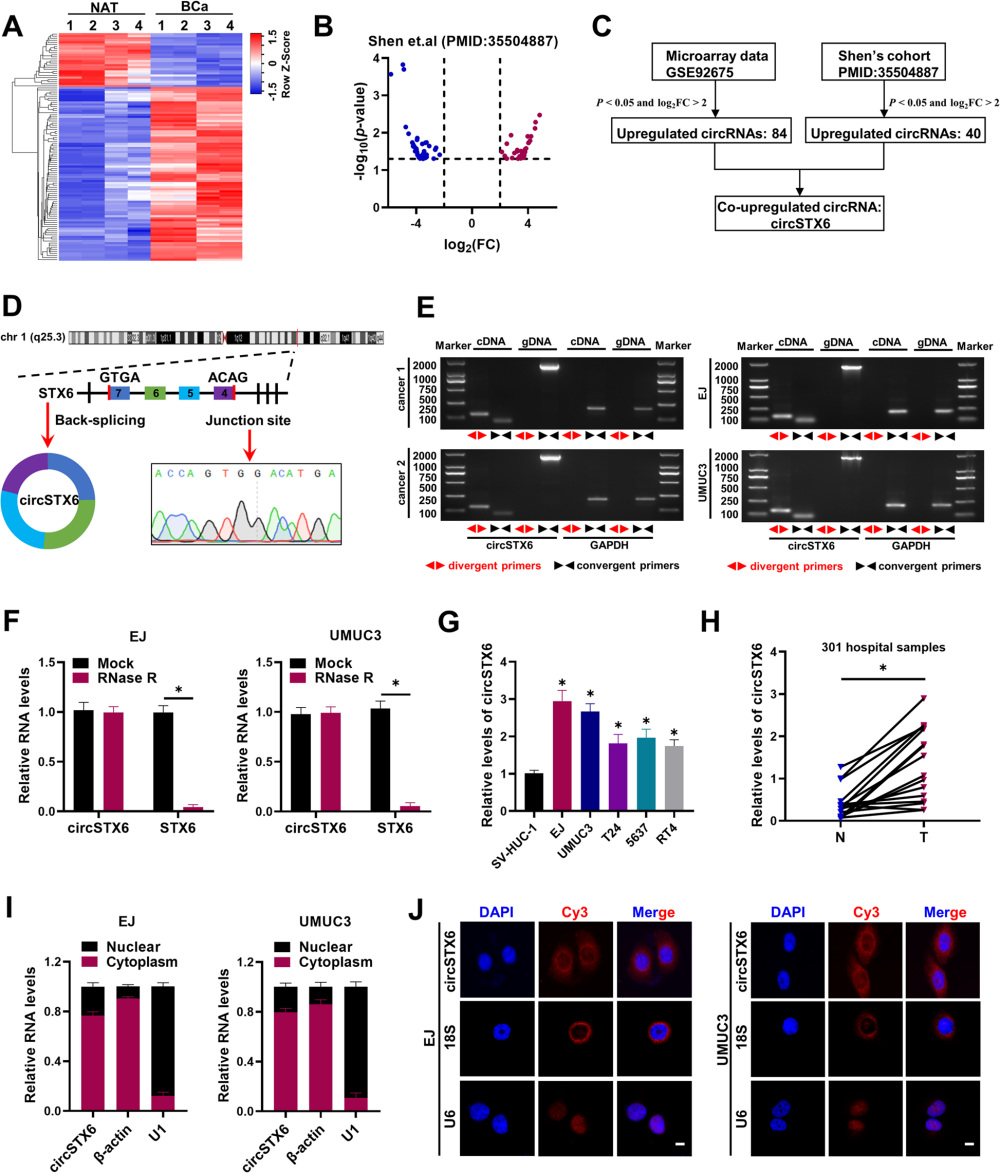
*CircSTX6* is verified and characterized in BCa cells and clinical samples. **A** Heatmap revealed the differentially expressed circRNAs in the four paired BCa tissues and NATs (GSE92675). **B** Volcano plots showed significantly different expression of circRNAs in paired five BCa tissues and NATs in research of Shen et al. |log2(FC)| > 2, *P* < 0.05. **C** Schematic diagram showed the screening of co-upregulated circRNA in both cohorts. **D** The back-splicing junction of *circSTX6* was identified by Sanger sequencing. The red arrow showed the “head-to-tail” splicing sites of *circSTX6*. **E** The existence of *circSTX6* was validated in two bladder cancer tissues and EJ, UMUC3 cell lines by RT-PCR and gel electrophoresis. Divergent primers amplified *circSTX6* in cDNA but not genomic DNA (gDNA). GAPDH was used as a control. **F** qRT-PCR analysis depicted the *circSTX6* and linear STX6 expression in BCa cells treated with or without RNase R. **G** qRT-PCR analysis revealed the levels of *circSTX6* in SV-HUC-1, EJ, UMUC3, T24, 5637 and RT4 cells. **H** qRT-PCR analysis revealed the levels of *circSTX6* in our 16 paired samples of BCa and NAT. N noncancerous adjacent tissues, T tumorous tissue. *P* values are calculated by paired two-sided t-test. **I** RNA levels of *circSTX6*, β-actin, and U1 in the nuclear and cytoplasmic fractions of bladder cancer cells. U1 was considered as a nuclear control and GAPDH was applied as positive controls in the cytoplasm. **J** RNA FISH analysis revealed that *circSTX6* was predominantly localized to the cytoplasm. Nuclei were stained with DAPI. Scale bar, 10 μm. The data are presented as the means ± S.D. of at least three independent experiments. *P < 0.05. *P* values are calculated by Student’s t test in **F** and one-way ANOVA in **G**

To identify the differentially expressed circRNAs in BCa, we mined the microarray profile (Fig. 1A) and the high-throughput RNA sequencing data (Fig. 1B) from two published researches [23, 24], and identified 84 and 40 upregulated circRNAs [log2(FC) > 2, P < 0.05] in BCa tissue respectively. Among them, only *circSTX6* (*has_circ_0007905*) was co-upregulated in both cohorts (Fig. 1C), which attracted our attention. Next, we examined that the physical circular structure of *circSTX6* was a 391-nt circRNA derived from exons 4, 5, 6 and 7 within the *STX6* locus, and the back-spliced junction site of which was amplified with divergent primers and validated by Sanger sequencing (Fig. 1D). However, head-to-tail splicing could be generated from trans-splicing or genomic rearrangements. Thus, we took several assays to rule out these possibilities [8]. Firstly, using cDNA and genomic DNA (gDNA) from BCa tissues and cells as templates, *circSTX6* was amplified only in cDNA groups by divergent primers rather than in the gDNA groups (Fig. 1E). Secondly, resistance to digestion with RNase R exonuclease also verified that *circSTX6* harbored a stable structure (Fig. 1F). As expected, BCa cells exhibited higher *circSTX6* expression level than SV-HUC-1 cells (Fig. 1G). In addition, *circSTX6* was significantly upregulated in our BCa tissues compared with paired normal adjacent tissues (NATs) (Fig. 1H). Next, we analyzed correlation between *circSTX6* expression and clinicopathologic features and found that high expression of *circSTX6* was positively associated with pathological stage, grade and muscle invasion in BCa patients (Table S1). We further detected the subcellular location of *circSTX6*. Nuclear and cytoplasmic fractionations indicated that *circSTX6* was mainly localized in the cytoplasm (Fig. 1I), as demonstrated by FISH for *circSTX6* (Fig. 1J). Taken together, these findings indicate that *circSTX6* is upregulated and mainly localized in the cytoplasm in BCa.

### EIF4A3-induced *circSTX6* promotes metastasis of BCa in vitro and in vivo

**Fig. 2:**
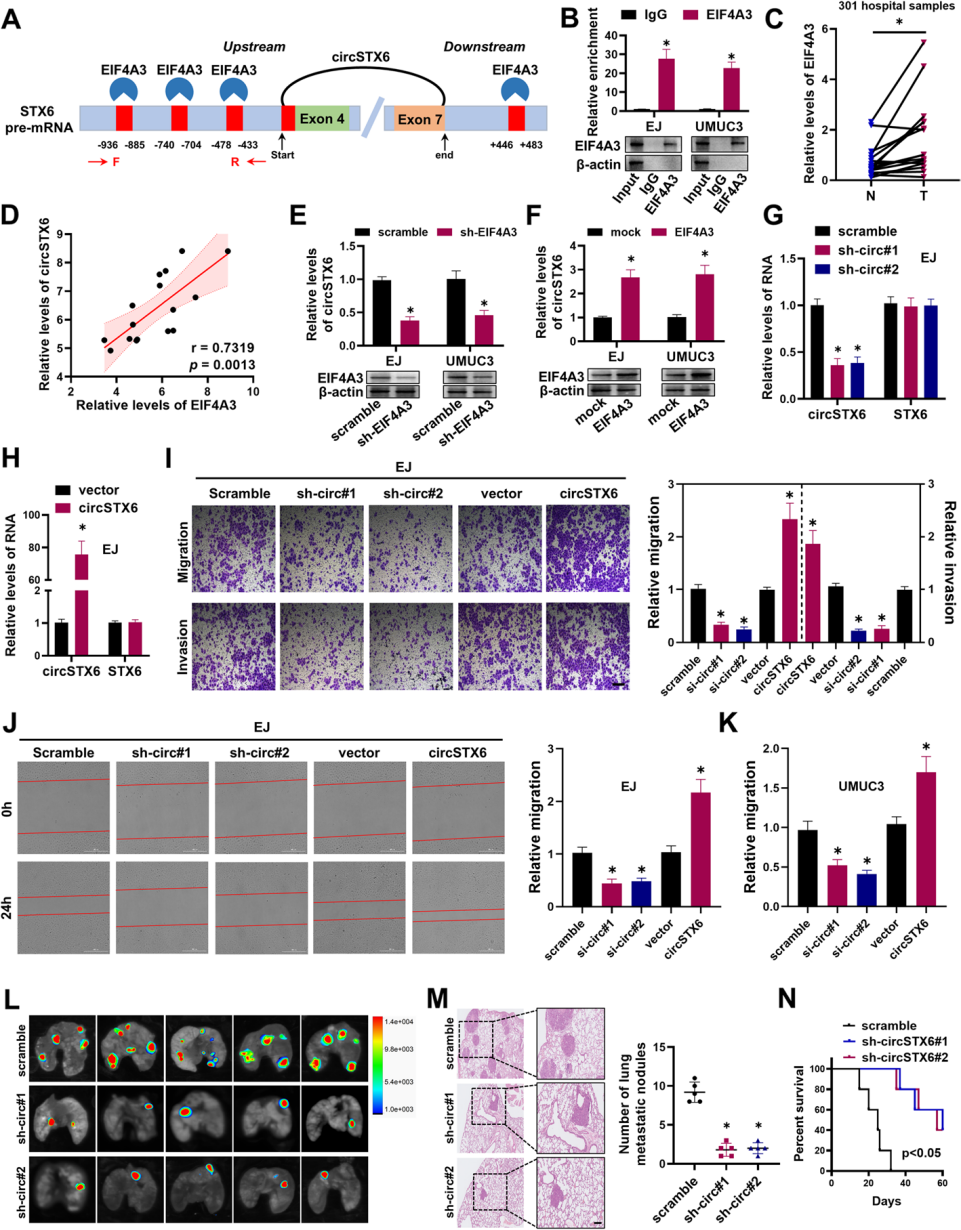
EIF4A3-induced *circSTX6* promotes metastasis of BCa in vitro and *in vivo.* **A** The binding sites for EIF4A3 in the flanking sequences of STX6 pre-mRNA were predicted using CircInteractome database. The primers were used to detect the levels of STX6 pre-mRNA. **B** RIP assay was performed to identify the putative binding sites of EIF4A3 in the STX6 pre-mRNA. IgG were used as the negative controls. **C** The relative expression of EIF4A3 in BCa samples and their para-cancer tissues were detected by qRT-PCR. *P* values are calculated by paired two-sided t-test. **D** The correlation between the expression of EIF4A3 and *circSTX6* in clinical BCa samples. **E-F** *CircSTX6* expression was detected in BCa cells after EIF4A3 down-regulation **(E)** or up-regulation **(F)** by qRT-PCR. **G** The knockdown efficiency of *circSTX6* in EJ cells was detected by qRT-PCR. **H** The overexpression efficiency of *circSTX6* in EJ cells was detected by qRT-PCR. **I** Representative and quantified results of the Transwell migration and invasion assays in EJ cells transfected with scramble, sh-circ#1, sh-circ#2, vector or circSTX6. Scale bar, 100 μm. **J-K** Cell migration capability of EJ and UMUC3 cells transfected with scramble, shcirc#1, shcirc#2, vector or circSTX6 was evaluated by wound healing assays. Scale bar, 400 μm. **L** Representative bioluminescence images showed the pulmonary metastasis focuses in the nude mice (*n* = 5 per group). **M** H&E staining was performed for histological confirmation of metastasizing tumor in lung. Scale bars, 200 μm. **N** Kaplan-Meier curves analysis showed the effect of knockdown *circSTX6* on the survival of the above nude mice. The data are presented as the means ± S.D. of at least three independent experiments. *P < 0.05. *P* values are calculated by Student’s t test in **B**, **E**, **F**, **H, I, J** and **K** and one-way ANOVA in **G**, **I, J, K** and **M**

The biogenesis of exon-derived circRNAs can be regulated by RNA-binding proteins (RBP) that binds to the flanking intron sequences of circRNAs [25–27]. Therefore, to further explore how *circSTX6* was upregulated in BCa, we searched the CircInteractome database to predict potential RBP sites in the flanking regions of *circSTX6*, and found that EIF4A3 had the most possible binding sites (Fig. 2A). EIF4A3 is a key component of the exon junction complex and plays an essential role in circRNAs cyclization and expression [28]. We hypothesized that the upregulation of *circSTX6* may be resulted from the increase of EIF4A3, which is involved in generating *circSTX6*. RIP assay confirmed that EIF4A3 indeed could bind to the upstream flanking sequence of *STX6* pre-mRNA (Fig. 2B). Moreover, the expression of EIF4A3 was upregulated in BCa tissues (Fig. 2C), and the level of *circSTX6* was significantly positively correlated with EIF4A3 expression (Fig. 2D). Inhibition of EIF4A3 led to a reduction of *circSTX6* (Fig. 2E). Conversely, overexpression of EIF4A3 increased the level of *circSTX6* (Fig. 2F). These data indicate that EIF4A3 binds to the *circSTX6* flanking intron sequences of *STX6* pre-mRNA to promote *circSTX6* formation.

To elucidate the functions of *circSTX6* in BCa, we designed two shRNAs to specifically target the backsplice junction region of *circSTX6* but not change linear *STX6* mRNA expression (Fig. 2G and Fig. S1A). Transwell assays revealed that silencing of *circSTX6* inhibited cell migration and invasion abilities of BCa cells (Fig. 2I and Fig. S1C). We also transfected a *circSTX6* overexpression plasmid that significantly promoted the expression of *circSTX6* and that it did not affect *STX6* mRNA levels (Fig. 2H and Fig. S1B). Overexpression of *circSTX6* significantly promoted the migration and invasion abilities of BCa cells (Fig. 2I and Fig. S1C). In addition, wound healing assays showed similar results that overexpression of *circSTX6* promoted cell migration, whereas silencing of *circSTX6* showed the reverse effects (Fig. 2J-K and Fig. S1D). On the other hand, CCK8 and EdU assays were performed, and the results indicated that overexpression or knockdown of *circSTX6* in BCa cells had no obvious effect on cell proliferation (Fig. S1E-F).

To investigate the effects of *circSTX6* on BCa metastasis in vivo, EJ cells stably transfected with sh-circ#1, sh-circ#2 or control vector were injected into nude mice via the tail vein to establish the lung metastasis model. Supporting the results obtained in vitro, as shown in Fig. 2L, knockdown of *circSTX6* decreased the number of pulmonary metastatic nodules. H&E staining demonstrated that mice with *circSTX6* depletion revealed smaller and fewer metastatic tumor nodules in the lungs (Fig. 2M). Furthermore, mice with low *circSTX6* expression had a favorable overall survival (Fig. 2N). Taken together, these findings imply that EIF4A3-induced *circSTX6* could play an oncogenic role to promote invasion and metastasis in BCa.

### *CircSTX6* serves as a *miR-515-3p* sponge

**Fig. 3:**
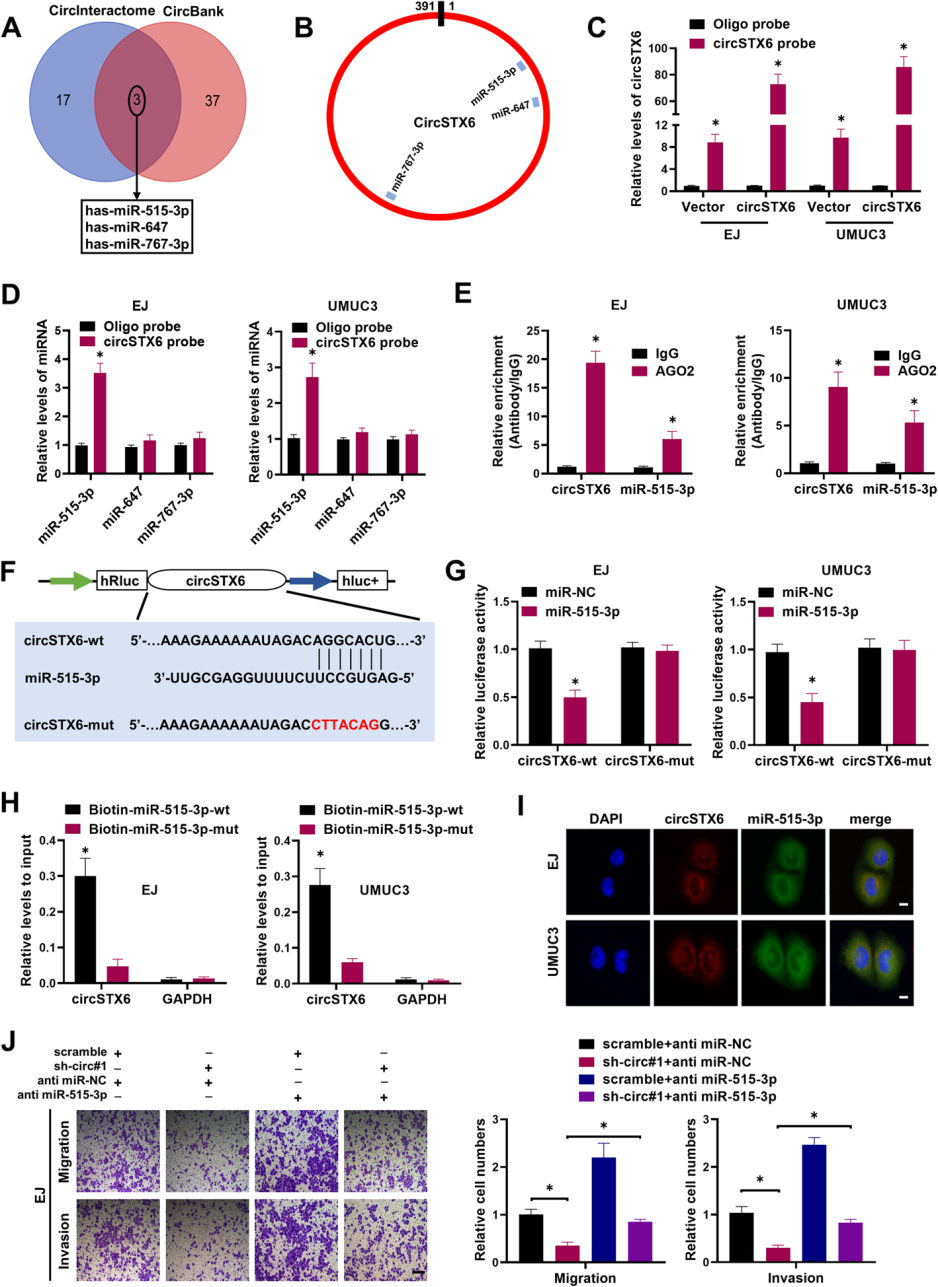
*CircSTX6* directly interacts with *miR-515-3p* in BCa. **A** The potential binding miRNAs with *circSTX6* were predicted by the databases of CircInteractome and CircBank. **B** Schematic diagram showed the putative binding sites of three miRNA candidates associated with *circSTX6*. **C** The efficiency of *circSTX6* probe were tested by RNA pulled down and qRT-PCR. **D** Relative levels of three miRNAs in EJ and UMUC3 lysates pulled down by *circSTX6* probe or oligo probe. **E** RIP assays were performed to detect the enrichment of *circSTX6* and *miR-515-3p* upon AGO2 antibody in BCa cells. **F** Schematic of circSTX6 wild-type (wt) and mutant (mut) luciferase reporter vectors. **G** Luciferase reporter assays revealed the relative luciferase activity of wild type or mutant circSTX6 in the miR-515-3p mimics or NC group. **H** The RNAs captured by biotinylated wild-type or mutant miR-515-3p BCa cells were quantified by qRT-PCR. **I** Co-localization of *circSTX6* and *miR-515-3p* in BCa cells was detected by FISH assay. Scale bar, 10 μm. **J** Transwell assays showed the metastasis in BCa cells stably transfected with scramble or sh-circ#1, and those co-transfected with anti-miR-NC or anti miR-515-3p. Scale bar, 100 μm. The data are presented as the means ± S.D. of at least three independent experiments. *P < 0.05. *P* values are calculated by Student’s t test in **C-E**, **G** and **H** and one-way ANOVA in **J**

Since circRNAs predominantly located in the cytoplasm were suggested to function as miRNA sponge [29, 30], we further explored whether *circSTX6* could bind to miRNAs. Three potential miRNAs associated with *circSTX6* were predicted by CircInteractome and CircBank databases (Fig. 3A) and the positions of putative binding sites in *circSTX6* were shown in Fig. 3B. Next, we investigated whether those candidate miRNAs could directly bind to *circSTX6*. The biotin-labeled *circSTX6* probe and oligo probe were designed and verified to pull down *circSTX6* in BCa cell lines, and the pull-down efficiency was enhanced in cells with stable *circSTX6* overexpression (Fig. 3C). Among the three candidate miRNAs, only *miR-515-3p* could be abundantly pulled down by *circSTX6* probe in both BCa cells (Fig. 3D). miRNA was reported to function as a RISC component by binding to AGO2 protein [31]. RIP assay confirmed that both *circSTX6* and *miR-515-3p* were markedly enriched in the precipitate obtained with the anti-AGO2 antibody (Fig. 3E).

To further confirm the sponge effect between *circSTX6* and *miR-515-3p*, dual-luciferase reporter assay revealed that the luciferase activity of *circSTX6-wt* but not *circSTX6-mut* obviously reduced in the miR-515-3p mimics group, compared with their NC group (Fig. 3F-G), suggesting that *circSTX6* could interact with *miR-515-3p*. Meanwhile, the enrichment of *circSTX6* in the captured fraction was much higher in biotin-miR-515-3p-wt group than in biotin-miR-515-3p-mut group (Fig. 3H), indicating that *miR-515-3p* could also bind to *circSTX6*. Moreover, FISH assays revealed that *circSTX3* and *miR-515-3p* were co-localized in the cytoplasm (Fig. 3I). Transwell assays further indicated that enforced expression of *miR-515-3p* significantly inhibited the migration and invasion of BCa cells (Fig. S2A-B). In contrast, the opposite results were obtained when the cells were transfected with *miR-515-3p* inhibitor (Fig. S2A-B). In addition, wound healing assays showed similar results (Fig. S2C-D). Furthermore, *miR-515-3p* inhibitor partially attenuated the reduction abilities of migration and invasion induced by *circSTX6* knockdown (Fig. 3J and Fig. S2E). Collectively, these results indicate that *circSTX6* might function as a competing endogenous RNA (ceRNA) through binding to *miR-515-3p*.

### *CircSTX6* inhibits *miR-515-3p*-mediated *SUZ12* degradation

**Fig. 4:**
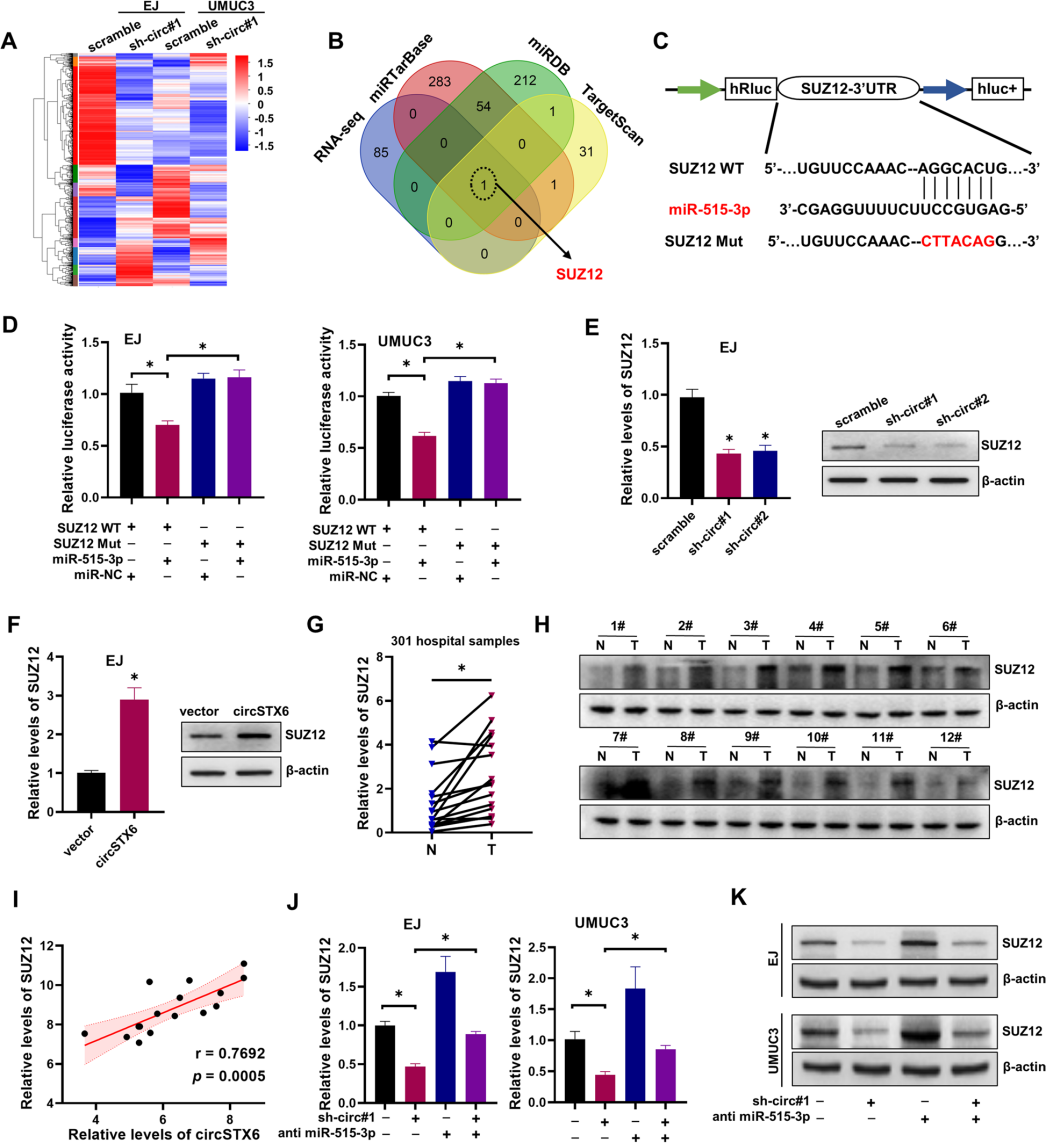
*CircSTX6* serves as a *miR-515-3p* sponge to upregulate SUZ12 expression. **A** Heat map showed the differentially expressed genes in EJ and UMUC3 cells after knockdown of *circSTX6*. **B** Venn diagram showed the genes detected by RNA-seq and overlapping analysis with the target mRNA of *miR-515-3p* predicted by miRTarBase, miRDB and TargetScan databases. **C** Schematic of SUZ12 3’ UTR wild-type (wt) and mutant (mut) luciferase reporter vectors. **D** The relative luciferase activities were analyzed in BCa cells co-transfected with miR-515-3p or miR-NC and luciferase reporter vectors SUZ12 3’UTR (WT) or SUZ12 3’UTR (Mut). **E-F** The mRNA and protein level of SUZ12 in EJ cells stably transfected scramble, sh-circ#1, sh-circ#2, vector or circSTX6. **G** qRT-PCR analysis revealed the levels of SUZ12 in our 16 paired samples of BCa and NAT. N noncancerous adjacent tissues, T tumorous tissue. *P* values are calculated by paired two-sided t-test. **H** The expression of SUZ12 was analyzed using western blot in our clinical samples. **I** The correlation between the transcript levels of *circSTX6* and SUZ12 in BCa tissues was analyzed (*n* = 16). **J-K** qRT-PCR and western blot were performed to evaluate the expression of SUZ12 in EJ and UMUC3 cells which were transfected with scramble or sh-circ#1, and those co-transfected with anti-miR-NC or anti miR-515-3p. The data are presented as the means ± S.D. of at least three independent experiments. *P < 0.05. *P* values are calculated by Student’s t test in **F** and one-way ANOVA in **D**, **E** and **J**

To elucidate the specific mechanism by which *circSTX6* exerts oncogene functions, EJ and UMUC3 cells stably transfected with *circSTX6* shRNA#1 were performed for gene expression profiling (Fig. 4A). Comprehensive analysis of the potential target genes of *miR-515-3p* using three bioinformatics algorithms including TargetScan, miRDB and miRTarbase with our RNA-seq results indicated that *SUZ12* might be regulated by *circSTX6* and *miR-515-3p* (Fig. 4B). Following insertion of wild-type and mutated *SUZ12* 3’UTR sequences into psiCHECK2.0 vectors and co-transfection with miR-515-3p, luciferase reports found that *miR-515-3p* could decrease the luciferase activity of wt 3’ UTR of *SUZ12* but not mut 3’ UTR of *SUZ12* (Fig. 4C-D). These results suggest that *miR-515-3p* could bind to the 3’-UTR of *SUZ12*. We then found that knockdown of *circSTX6* inhibited the *SUZ12* mRNA and protein expression (Fig. 4E and Fig. S3A). In contrast, overexpression of *circSTX6* completely had the opposite effect (Fig. 4F and Fig. S3A). The *SUZ12* mRNA level was significantly higher in BCa tissues compared with NATs according to the TCGA dataset (Fig. S3B) and our BCa tissues (Fig. 4G). We also found that the protein level of SUZ12 was upregulated in bladder cancer tissues (Fig. 4H). Importantly, the level of *SUZ12* was positively correlated with *circSTX6* expression (Fig. 4I). The reduction of *SUZ12* mRNA and protein levels caused by sh-circ#1 transfection was partially reversed by transfection with a *miR-515-3p* inhibitor (Fig. 4J-K). Furthermore, enforced expression of SUZ12 could significantly reverse the decrease of SUZ12 expression caused by *circSTX6* knockdown (Fig. S3C). Taken together, these data indicate that *circSTX6* could sponge with *miR-515-3p* to promote the expression of SUZ12.

### Silencing of SUZ12 reverses the *circSTX6*-mediated oncogenic effect

**Fig. 5:**
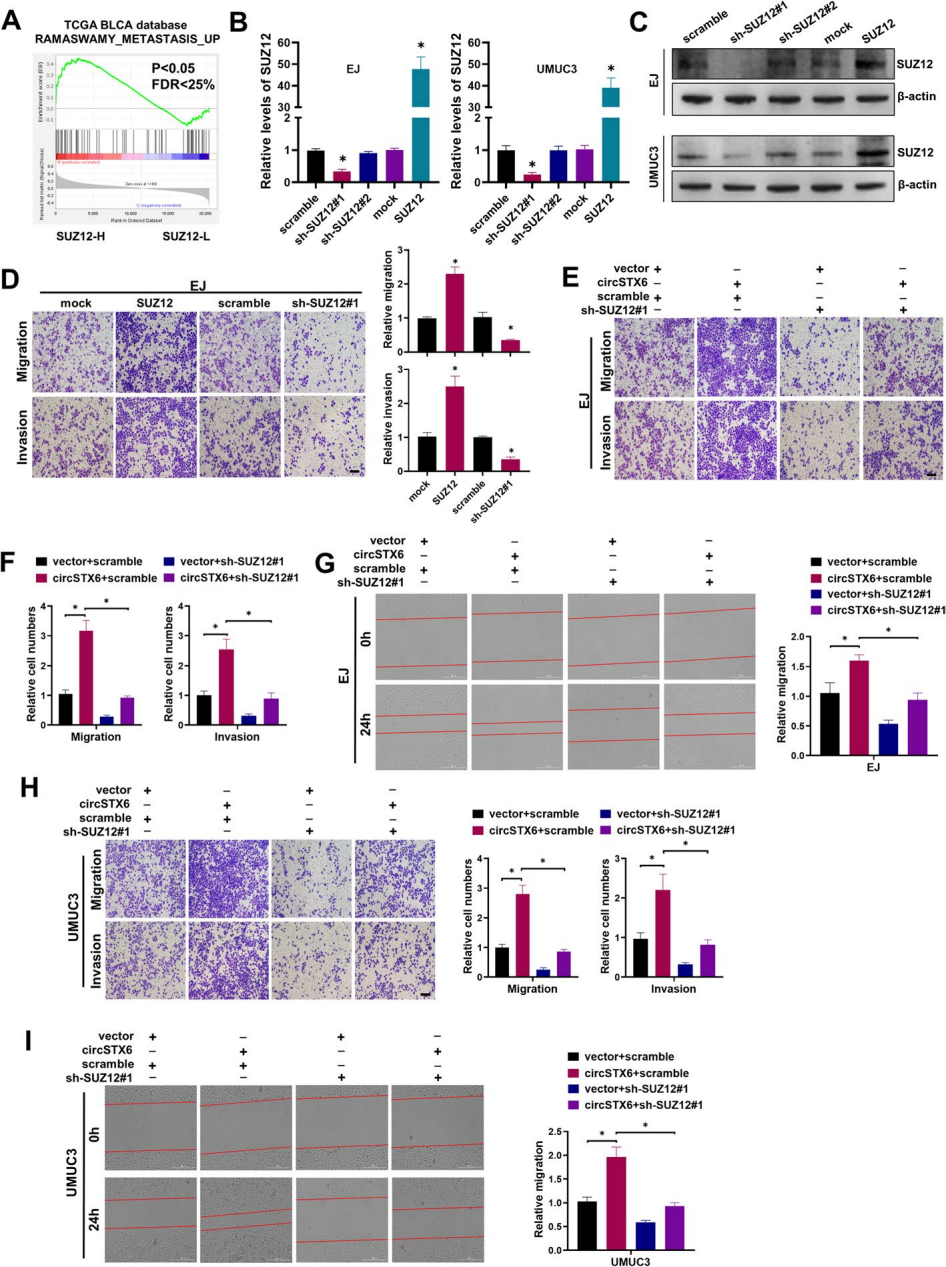
*CircSTX6* promotes BCa metastasis via the *miR-515-3p*/SUZ12 axis. **A** GSEA plot showed that the levels of *SUZ12* expression were positively correlated with the metastasis ability. **B-C** The knockdown and overexpression efficiencies of SUZ12 were verified by qRT-PCR and western blot assays. **D** Representative and quantified results of the Transwell migration and invasion assays in the mock, SUZ12, scramble or sh-SUZ12#1 group. Scale bar, 100 μm. **E-F** Representative and quantified results of the Transwell migration and invasion assays in EJ cells transfected with vector or circSTX6, and those co-transfected with scramble or sh-SUZ12#1 plasmid. Scale bar, 100 μm. **G** Wound healing assays showed the migration abilities in EJ cells transfected with vector or circSTX6, and those co-transfected with scramble or sh-SUZ12#1 plasmid. Scale bar, 400 μm. **H** Representative and quantified results of the Transwell migration and invasion assays in UMUC3 cells transfected with vector or circSTX6, and those co-transfected with scramble or sh-SUZ12#1 plasmid. Scale bar, 100 μm. **I** Wound healing assays showed the migration abilities in UMUC3 cells transfected with vector or circSTX6, and those co-transfected with scramble or sh-SUZ12#1 plasmid. Scale bar, 400 μm. The data are presented as the means ± S.D. of at least three independent experiments. *P < 0.05. *P* values are calculated by Student’s t test in **B**, **D** and one-way ANOVA in **B**, **F-I**

To evaluate the effects of SUZ12 on BCa phenotypes, we performed Gene Set Enrichment Analysis (GSEA) analysis to compare gene profiles of BCa samples with high and low *SUZ12* expression using TCGA BLCA database. The results indicated that *SUZ12*-high BCa samples were significantly enriched in gene sets related to metastasis, which is consistent with the promoting role of *circSTX6* in BCa metastasis (Fig. 5A). To study the functions of SUZ12, the stably transfected cell lines with overexpression and knockdown of SUZ12 were constructed (Fig. 5B-C). Enforced expression of SUZ12 significantly increased invasion and migration ability of BCa cells, while knockdown of SUZ12 exhibited contrary effects (Fig. 5D and Fig. S4A-C). To determine whether *circSTX6* functions as an oncogene via SUZ12, we performed rescue experiments. The results showed that knockdown of SUZ12 could reverse the promotion of cell migration and invasion mediated by *cirSTX6* overexpression (Fig. 5E-I). Therefore, these data demonstrate that *circSTX6* acts as a sponge for *miR-515-3p* to promote the expression of SUZ12 and enhances BCa metastasis.

### *CircSTX6* interacts with the PABPC1 protein in bladder cancer

**Fig. 6:**
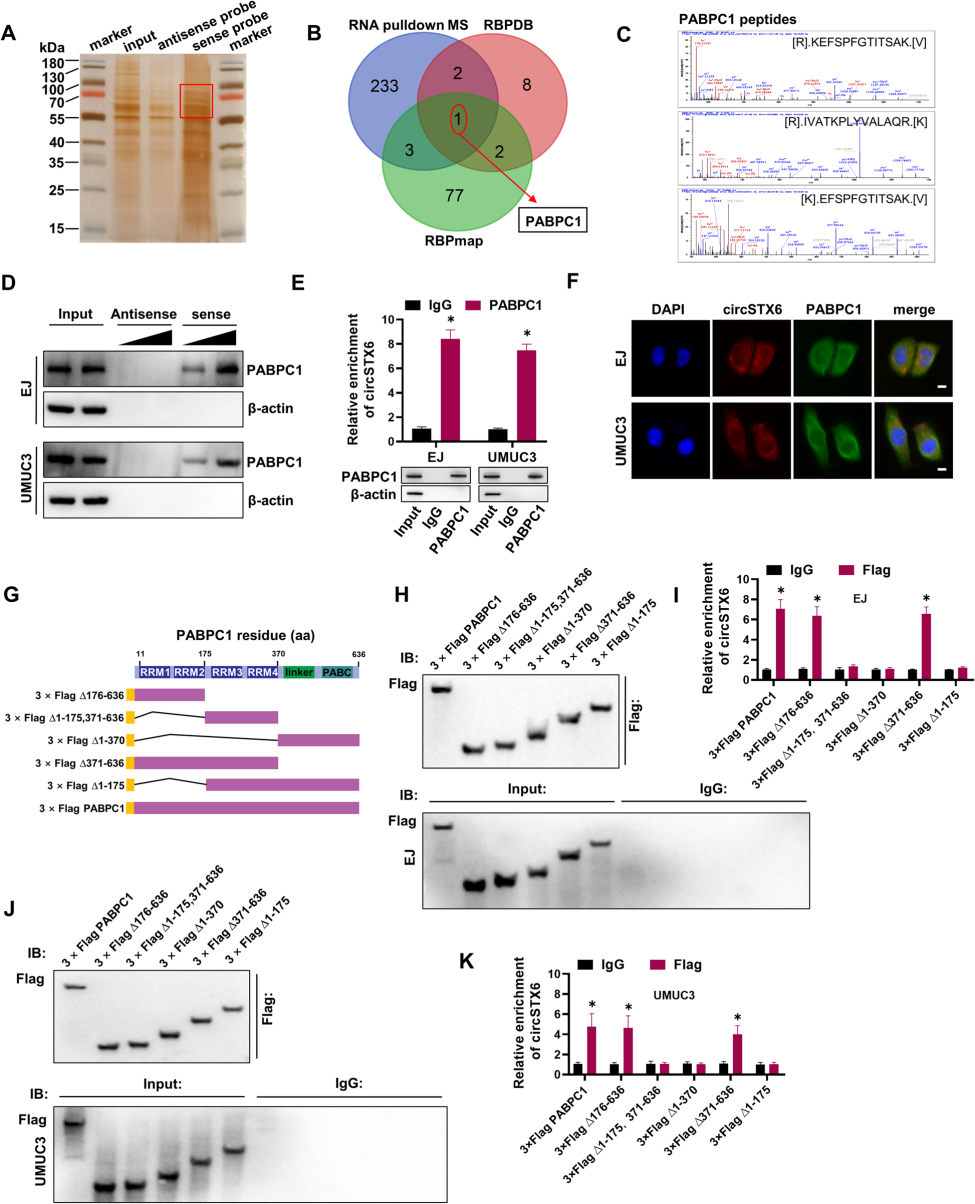
*CircSTX6* interacts with the PABPC1 protein in BCa. **A** Silver staining depicted the proteins that interact with *circSTX6*. Biotin-labeled sense or antisense *circSTX6* probes were used for RNA protein pull-down against EJ cells lysates. **B** Venn diagram demonstrated the overlapping of the interacting RBPs of *circSTX6* determined by Mass spectrometry and predicted by RBPmap and RBPDB databases. The 239 proteins with molecular masses of 55–100 kDa that were only pulled down by sense probe were screened. **C** Mass spectrometry assay depicted the PABPC1 peptides pulled down by *circSTX6* sense probes. **D** Western blot assay showed the PABPC1 protein pulled down by biotin-labeled *circSTX6* antisense or sense probes from the lysates of BCa cells. **E** RIP assays showed the association of PABPC1 and *circSTX6* (upper). The precipitate was subjected to western blot with the antibodies against PABPC1 (lower). IgG was used as the negative control. **F** The subcellular location of PABPC1 and *circSTX6* in BCa cells was evaluated by IF and FISH assay. Nuclei were stained with DAPI. Scale bar, 10 μm. **G** The schematic structures revealed the full-length and truncations plasmids for PABPC1. **H** Full-length or truncations of Flag-tagged recombinant PABPC1 proteins in EJ cells were validated by western blot analysis. **I** Relative enrichment of *circSTX6* levels was detected by RIP and qRT-PCR assays in EJ cells transfected with Flag-tag plasmids of PABPC1 full-length or truncations. **J** Full-length or truncations of Flag-tagged recombinant PABPC1 proteins in UMUC3 cells were validated by western blot analysis. **K** Relative enrichment of *circSTX6* levels was detected by RIP and qRT-PCR assays in UMUC3 cells transfected with Flag-tag plasmids of PABPC1 full-length or truncations. The data are presented as the means ± S.D. of at least three independent experiments. *P < 0.05. *P* values are calculated by Student’s t test in **E**, **I** and **K**

Since cytoplasmic circRNAs could also perform their functions by directly binding to specific RBPs [32], we next designed a biotin-labeled *circSTX6* probe and performed RNA pulldown assays to explore its protein binding role. The proteins pulled down through biotin-labeled *cirSTX6* antisense or sense probes were detected by Western blotting and silver staining (Fig. 6A). Comprehensive analysis of *circSTX6* potential binding proteins from RBPmap and RBPDB database with our mass spectrometry results indicated that PABPC1 could potentially interact with *circSTX6* (Fig. 6B). The identified peptides of PABPC1 from MS assay were shown in Fig. 6C. Validating biotin-labeled RNA pulldown assay further indicated that *circSTX6* sense probe was able to dose dependently interact with PABPC1 (Fig. 6D). In addition, the interaction between *circSTX6* and PABPC1 was further validated by RIP assays (Fig. 6E). FISH-immunofluorescence assays found that *circSTX6* co-localized with PABPC1 in the cytoplasm (Fig. 6F), indicating that *circSTX6*/PABPC1 formed an RNA-protein complex in BCa cells. Accumulating evidence indicates that PABPC1 protein consists of four non-identical RNA recognition motifs (RRMs) followed by a linker region and the eRF3-binding PABC domain, and the domains of RRM1-2 exhibit highly RNA affinity than RRM3-4 [33, 34]. To delineate which domain of PABPC1 contributes to the interaction with *circSTX6*, we constructed six Flag-tagged vectors encoding truncations of PABPC1 (Fig. 6G). Consequently, RIP assays revealed that removal of the RRM1-2 of PABPC1 abolished its association with *circSTX6*, suggesting that the region between RRM1 and RRM2 of PABPC1 specifically bound to *circSTX6* (Fig. 6H-K). In summary, these results demonstrate that *circSTX6*/PABPC1 forms an RNA-protein complex through the RRM1 and RRM2 domains of PABPC1.

### *CircSTX6*/PABPC1 complex promotes the stability of *SUZ12* mRNA

**Fig. 7:**
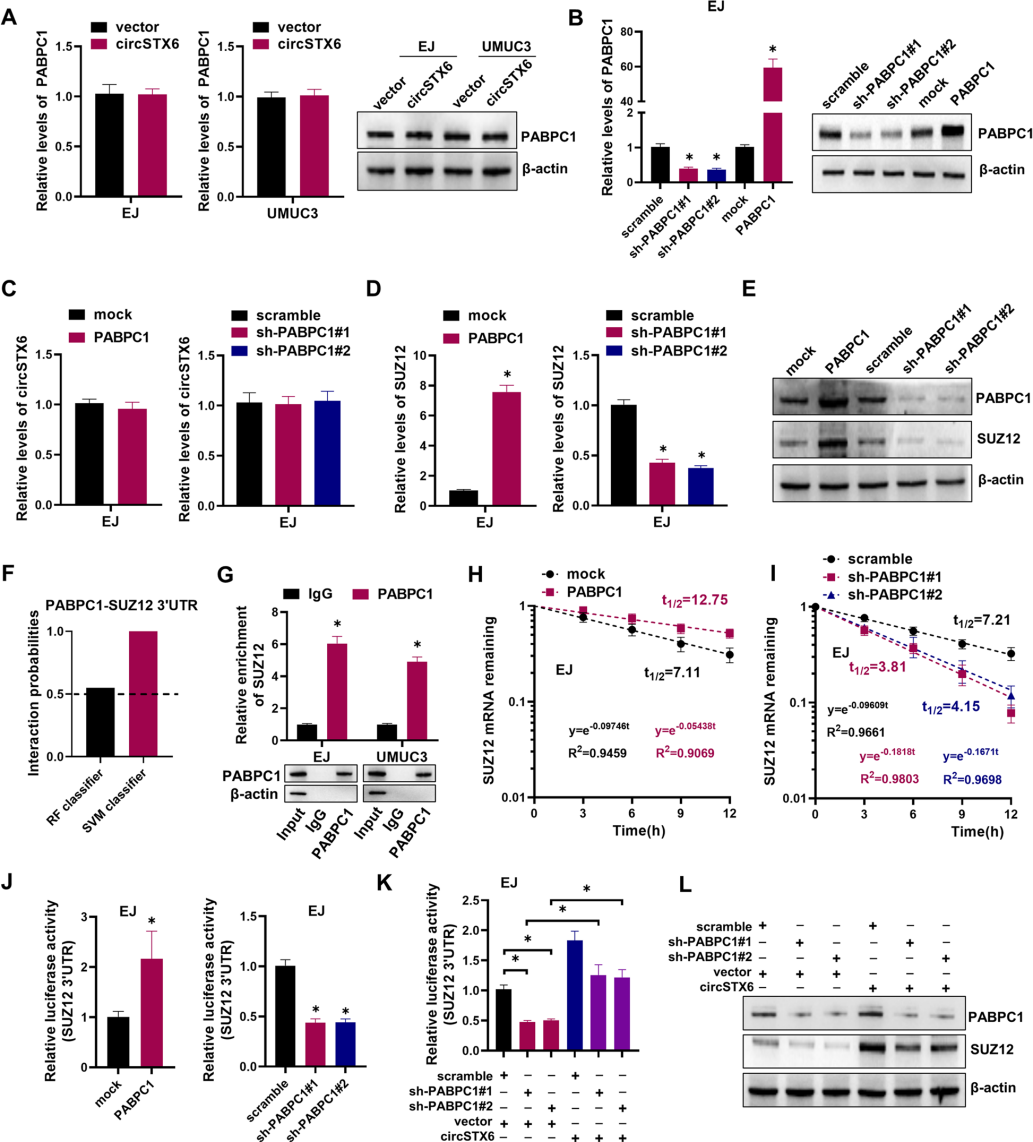
*CircSTX6*/PABPC1 complex promotes the stability of *SUZ12* mRNA. **A** qRT-PCR (left) and western blot (right) assays showed the expression of PABPC1 in BCa cells transfected with circSTX6 plasmids. **B** The efficiency of PABPC1 knockdown or overexpression in EJ cells was verified by qRT-PCR (left) and western blot (right). **C** qRT-PCR assay showed the expression of *circSTX6* in EJ cells transfected with the indicated plasmids. **D-E** qRT-PCR **(D)** and western blot **(E)** assays showed the expression of SUZ12 in EJ cells transfected with the indicated plasmids. **F** The interaction probabilities of PABPC1 with 3′-UTR of *SUZ12* mRNA were predicted by the RNA-Protein interaction prediction (RPISeq) website. Predictions with probabilities > 0.5 were considered “positive”, indicating that the corresponding RNA and protein are likely to interact. **G** RIP assays showed the association of PABPC1 and *SUZ12* (upper). The precipitate was subjected to western blot with the antibodies against PABPC1 (lower). IgG was used as the negative control. **H-I** RNA stability assays revealed the remaining levels of *SUZ12* mRNA in EJ cells transfected with overexpression plasmids of PABPC1 **(H)** or shRNA specifically targeting PABPC1 **(I)**, respectively. **J** Luciferase activities of *SUZ12* 3’UTR were measured after overexpression or knockdown of PBAPC1 in EJ cells, respectively. **K** Luciferase activities of *SUZ12* 3’UTR were measured in EJ cells transfected with scramble, sh-PABPC1#1, sh-PABPC1#2, and those co-transfected with vector or circSTX6 plasmid. **L** Western blot assays showed the expression of SUZ12 in BCa cells transfected with scramble, sh-PABPC1#1, sh-PABPC1#2, and those co-transfected with vector or circSTX6 plasmid. The data are presented as the means ± S.D. of at least three independent experiments. *P < 0.05. *P* values are calculated by Student’s t test in **B**, **D**, **G** and **J** and one-way ANOVA in **B**, **D**, **J** and **K**

Previous studies have demonstrated that circRNAs could regulate the expression of its binding RBPs [35, 36]. However, *circSTX6* did not affect mRNA and protein levels of PABPC1 (Fig. 7A). Given that PABPC1 is essential for RNA stability [37, 38] and *circSTX6* could interact with PABPC1, we examined whether PABPC1 regulated the expression of *circSTX6*. As shown in Fig. 7B-C and Fig. S5A-B, the levels of *circSTX6* did not significantly change by PABPC1. On the other hand, since SUZ12 as downstream target of *circSTX6*, we were very curious whether PABPC1 cooperated with *circSTX6* to regulate the expression of SUZ12. It showed that enforced expression of PABPC1 efficiently upregulated the mRNA and protein expression of SUZ12 (Fig. 7D-E and Fig. S5C). Conversely, knockdown of PABPC1 had opposite effects (Fig. 7D-E and Fig. S5C). The interaction between PABPC1 and 3’UTR of *SUZ12* was further predicted by the RNA-Protein interaction prediction (RPISeq) website. The scores of RF classifier and SVM classifier were both over 0.5 (Fig. 7F), indicating that PABPC1 had a great possibility of interaction with 3’UTR of *SUZ12* mRNA. Next, we performed RIP assay and confirmed that PABPC1 indeed bound to the *SUZ12* mRNA (Fig. 7G). RNA stability assays found that the half-life of *SUZ12* mRNA was dramatically increased in PABPC1 overexpression and reduced in PABPC1 knockdown (Fig. 7H-I). Moreover, ectopic expression of PABPC1 significantly increased the luciferase activity of *SUZ12* 3’-UTR reporter, whereas knockdown of PABPC1 decreased the luciferase activity of *SUZ12* 3’-UTR reporter (Fig. 7J). Additionally, overexpression of *circSTX6* reversed the reduction of the luciferase activity of *SUZ12* 3’-UTR reporter induced by PABPC1 knockdown (Fig. 7K). Meanwhile, silencing of PABPC1 decreased the protein level of SUZ12, which was reversed by ectopic expression of *circSTX6* (Fig. 7L). Taken together, these findings indicate that *circSTX6* enhances the mRNA stability of *SUZ12* through forming a *circSTX6*/PABPC1/*SUZ12* RNA-protein ternary complex.

### *CircSTX6*-mediated DNA damage repair promotes CDDP resistance in BCa cells

**Fig. 8:**
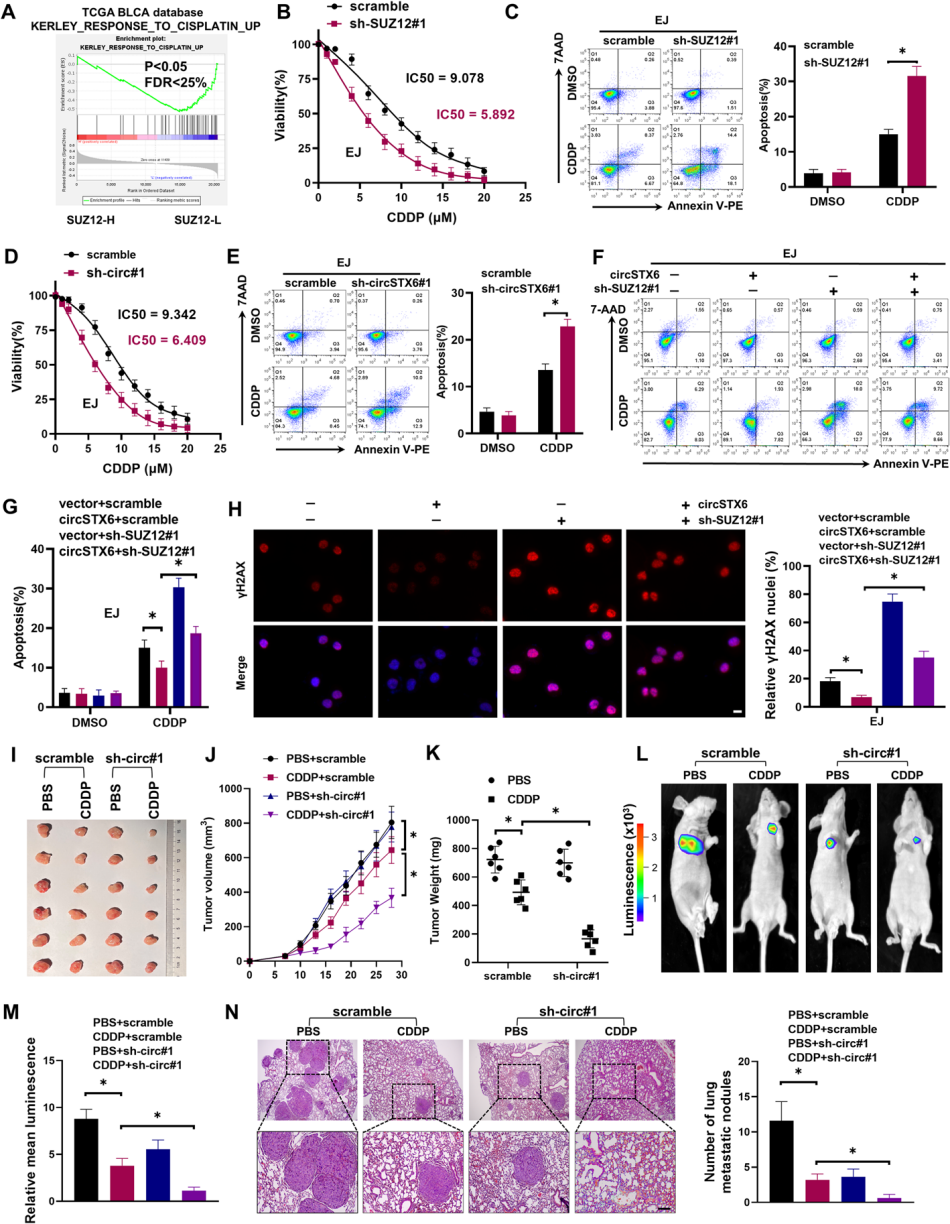
*CircSTX6* improves the chemosensitivity of BCa to CDDP. **A** GSEA plot showed that the levels of *SUZ12* expression were negatively correlated with the CDDP chemosensitivity. **B** Determination of IC50 values for CDDP treatment 24 h in EJ cells which were stably transfected with scramble or sh-SUZ12#1. **C** Flow cytometry apoptosis assays were used to assess apoptosis rate of EJ cells stably transfected with sh-SUZ12#1 upon CDDP treatment. **D** Determination of IC50 values for CDDP treatment 24 h in EJ cells which were stably transfected with scramble or sh-circ#1. **E** Flow cytometry apoptosis assays were used to assess apoptosis rate of EJ cells stably transfected with sh-circSTX6 after CDDP treatment. **F-G** Flow cytometry assay revealed the CDDP effect in EJ cells stably transfected with vector or circSTX6, and those co-transfected with scramble or sh-SUZ12#1. **H** Immunofluorescence staining showed the expression of γH2AX under CDDP treatment conditions in the indicated cells. The scale bar indicates 20 μm. **I** Representative images of xenograft tumors of circSTX6-knockdown group and their control group after being treated with either CDDP or PBS. **J-K** The tumor growth curves were drawn and tumor weight was measured. **L-M** Representative images of bioluminescence and histogram analysis of bioluminescence intensity in the indicated cell groups (*n* = 5 per group). **N** H&E staining was performed for histological confirmation of metastasizing tumor in lung. Scale bars, 200 μm. The data are presented as the means ± S.D. of at least three independent experiments. *P < 0.05. *P* values are calculated by Student’s t test in **C** and **E** and one-way ANOVA in **G**, **H**, **K**, **M** and **N**

Although promising results have been observed to inhibit BCa metastasis by inhibiting *circSTX6*, these effects focus on the basic biological characteristics of tumors. However, during clinical practice, CDDP chemoresistance remains a major challenge for BCa patients. Previous studies have demonstrated that SUZ12 plays an essential role in CDDP resistance in BCa cells [39]. Moreover, based on mRNA expression data from the TCGA BLCA, GSEA enrichment indicated that the expression level of *SUZ12* is negatively associated with the sensitivity to CDDP (Fig. 8A). As determined by CCK8 assay, the IC50 value of CDDP was decreased after knockdown of SUZ12, and was enhanced upon overexpression of SUZ12 in BCa cells (Fig. 8B and Fig. S6A). Importantly, silencing of SUZ12 increased the sensitivity of BCa cells to CDDP (Fig. 8C and Fig. S6B). Since *circSTX6* could promote the expression of SUZ12 through sponging *miR-515-3p* and binding with PABPC1. We sought to determine whether *circSTX6* could regulate the sensitivity to CDDP. Our results showed that IC50 value of CDDP was decreased when *circSTX6* was knocked down, and increased when *circSTX6* was overexpressed (Fig. 8D and Fig. S6C). Similarly, knockdown of *circSTX6* promoted the chemosensitivity of BCa cells to CDDP (Fig. 8E and Fig. S6D). Importantly, silencing of SUZ12 significantly rescued the decrease of CDDP chemosensitivity in BCa cells induced by overexpression of *circSTX6* (Fig. 8F-G). Furthermore, silencing of *circSTX6* had no effect on the ability of sphere formation (Fig. S6E), but promoted the expression of γH2AX upon CDDP treatment (Fig. S6F-G). SUZ12 knockdown also increased the expression of γH2AX (Fig. S6H-I). SUZ12 deletion reversed the reduction of DNA damage levels in BCa cells induced by *circSTX6* overexpression (Fig. 8H). To further define whether *circSTX6* was effective against acquired CDDP resistance in BCa, we performed experiments with a CDDP-resistant EJ cell lines (EJ-CDDP). In comparison with the parental EJ cell lines, the EJ-CDDP exhibited markedly increased *circSTX6* expression (Fig. S6J). As shown in Fig. S6K-M, EJ-CDDP resistant cells exhibited a high level of resistance to CDDP, and knockdown of *circSTX6* promoted the sensitivity of EJ-CDDP cells to CDDP. Importantly, we further confirmed that overexpression of *circSTX6* significantly decreased the EJ-CDDP cells apoptosis upon CDDP treatment, which could be reversed by silencing of SUZ12 (Fig. S6N).

**Fig. 9:**
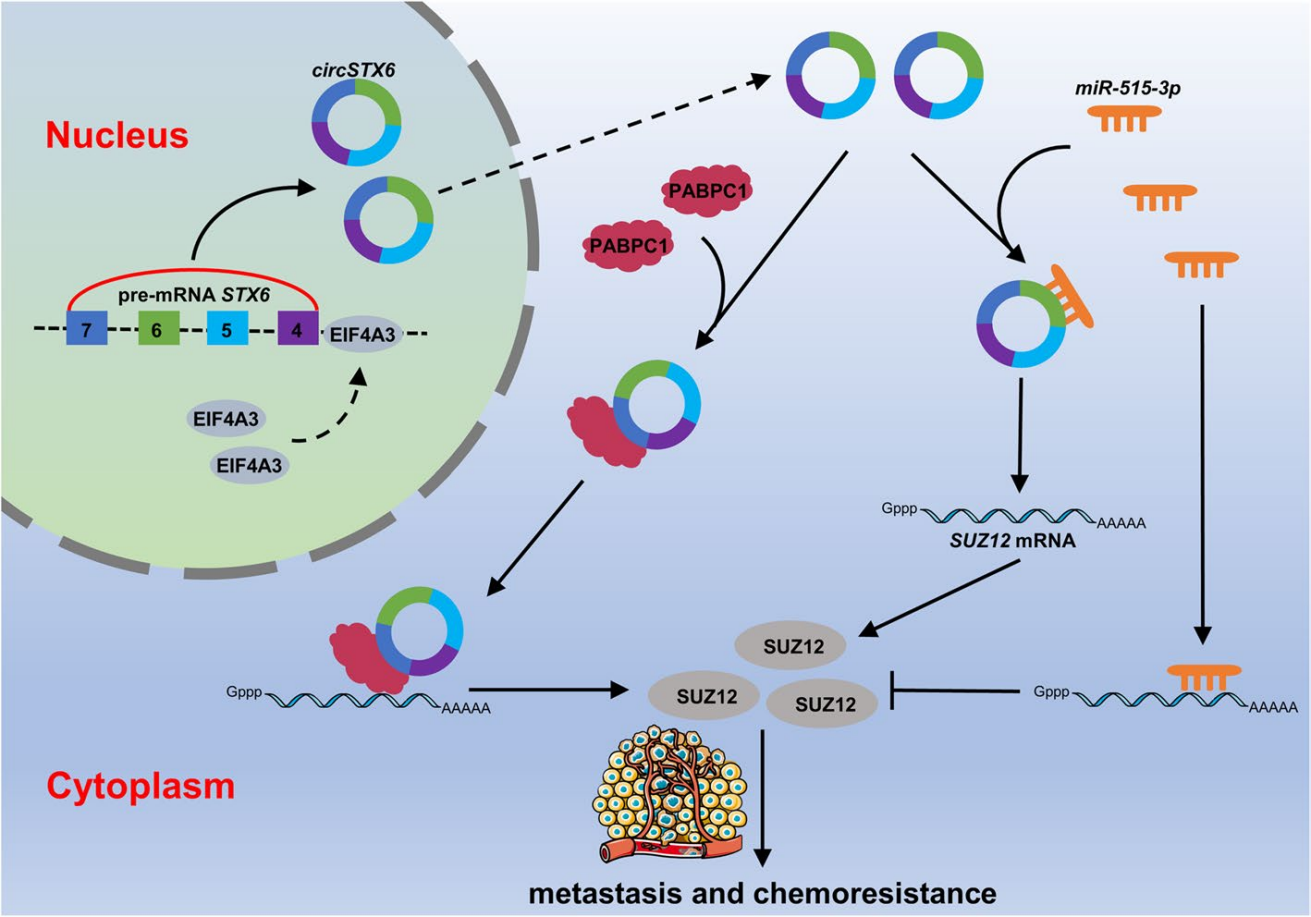
Schematic diagram describes the mechanism by which *circSTX6* promotes metastasis and chemoresistance of BCa through rehulation of SUZ12 by sponging *miR-515-3p* and interacting with PABPC1, respectively

 To determine whether *circSTX6* is an alternative therapeutic target that could improve the chemosensitivity in vivo. EJ cells stably transfected with sh-circ#1 or scramble plasmids were injected into BALB/c nude mice subcutaneously, followed by intraperitoneal PBS or CDDP treatment. Supporting the results obtained in vitro, knockdown of *circSTX6* had no effect on the volumes and weights of xenograft tumors, but improved the sensitivity of BCa tumors to CDDP (Fig. 8I-K). In addition, tumor metastasis model indicated that knockdown of *circSTX6* could effectively inhibit BCa cell metastasis upon CDDP treatment (Fig. 8L-N). Taken together, these findings indicate that *circSTX6* could promote the metastasis and CDDP resistance of BCa cells (Fig. 9), highlighting that targeting *circSTX6* provides a promising therapeutic strategy for CDDP-resistant BCa patients.

## Discussion

Bladder cancer is the most common malignancy of urinary tract with high morbidity and mortality rates [40], with a gradually increasing incidence these years in China [41]. CDDP-based chemotherapy is a most predominant chemotherapeutic strategy for most cancers, including BCa. However, resistance to CDDP agents, either intrinsic or acquired, is the main cause of the unfavorable prognosis of BCa patients currently [42]. Several circRNAs are crucial in regulating the CDDP chemotherapy sensitivity of BCa. For instance, our previous studies demonstrated that *circ0008399* could promote CDDP resistance by promoting the assembly of m^6^A methyltransferase complex in BCa [15]. Another study reported that Cdr1as could improve the CDDP chemosensitivity of BCa through the *Cdr1as*/*miR-1270*/APAF1 axis [43]. Herein, we uncovered that ectopic expression of *circSTX6* promoted CDDP resistance in vitro and in vivo, whereas inhibition of *circSTX6* could re-sensitize the CDDP-resistant BCa cells to CDDP. Interfering of *circSTX6* combined with CDDP significantly increased therapeutic outcomes, representing a promising therapeutic target for chemoresistant BCa. Previous studies have indicated that several factors are associated with CDDP resistance, including ATP-binding cassette (ABC) transporters, cancer stem cells (CSCs), epithelial-mesenchymal transition (EMT) and DNA damage repair [44, 45]. In our study, we have been confirmed that knockdown of *circSTX6* could facilitate the expression of γH2AX upon CDDP treatment. SUZ12 deletion reversed the reduction of γH2AX level induced by *circSTX6* overexpression. Therefore, we speculated that *circSTX6* could promote CDDP resistance through facilitating DNA damage repair. However, the potential mechanisms of *circSTX6*-mediated DNA damage repair upon CDDP treatment still remains to be further investigated.

Bladder tumors that invade the muscle layer are referred to as muscle-invasive bladder cancer (MIBC), which have a higher propensity to spread to lymph nodes and other organs. Multiple independent studies have demonstrated that lymphatic metastasis is a key prognostic factor in bladder cancer, accompanied by a reduction of the average 5-year survival rate to merely 18.6% [46]. Previous study has illustrated that lymphangiogenesis, which means the formation of new lymphatic vessels from the preexisting lymphatics, promotes the process of lymphatic metastasis [47]. Therefore, uncovering the mechanisms involved in lymphatic metastasis of BCa and exploring novel therapeutic strategies targeting this condition are urgently needed. It has been reported that ETV4 promotes TANs-mediated lymphangiogenesis and lymph node metastasis of BCa by regulating TANs infiltration [46]. In addition, HSF1-PRMT5-WDR5 axis plays key roles in the lymphatic metastasis of BCa, and targeting HSF1 by KRIBB11 is a multipotent and promising therapeutic strategy for BCa patients with lymphatic metastasis [48]. In our study, we have confirmed that *circSTX6* could promote distant lung metastasis in BCa in vivo. However, considering that BCa often exhibits lymphatic metastasis first. Whether *circSTX6* could regulate lymphangiogenesis and lymphatic metastasis of BCa still need to be further clarified.

Increasing evidence have indicated that the biogenesis of circRNAs is regulated by specific *cis*-regulatory elements and *trans*-acting factors [49]. Notably, certain RBPs also play an important role in the formation of circRNAs [50]. EIF4A3 functions as an RBP mainly localized in the nucleus, and is reported to bind RNA and form exon junction complexes, which is widely involved in exon splicing [51]. Recent studies have shown that EIF4A3 facilitates *circMMP9* and *circARHGAP29* expression by binding with the flank sequence of their parental pre-mRNA [52, 53]. In this study, we confirmed that EIF4A3 combined with the upstream flanking sequences of *circSTX6* pre-mRNA, which could promote the expression of *circSTX6*. Besides, Chan et al. have revealed that EIF4A3 could mediate the nuclear export of spliced RNA [51]. For example, EIF4A3 induces the cytoplasmic export of *circARHGAP29* through binding with the back-spliced junction site and the downstream flanking sequence of *circARHGAP29* in docetaxel-resistant PCa [53]. However, whether the high expression of *circSTX6* in cytoplasm was partially through the EIF4A3 dependent cytoplasmic export should be further investigated.

The mechanism by which circRNAs exert their functions is first described as miRNA sponges [8]. *CDR1as*, a classical circRNA, harboring 63 binding elements for *miR-7*, can function as miRNA sponges to regulate *miR-7* target mRNA expression. Thereafter, the functions of circRNAs as a “miRNA sponges” have been discovered to participate in many biological processes. In addition, the importance of circRNA-miRNA interactions is also reported in various human cancers, including BCa. *CircHIPK3* absorbs *miR-7* to promote the expression of proto-oncogenes in colorectal cancer [54]. *CircMTO1* suppresses hepatocellular carcinoma cell progression by sponging oncogenic *miR-9* to promote p21 expression [55]. *CircRIP2* targets *miR-1305* to promote Tgf-β2/smad3 pathway, while *circITCH* sponges *miR-17* and *miR-224* to enhance p21 and PTEN expression in BCa [56, 57]. Herein, we demonstrated that *circSTX6* absorbed *miR-515-3p*, and in turn promoted the expression of SUZ12. Nevertheless, the roles of *miR-515-3p* are not yet known in BCa. We first discovered that *miR-515-3p* inhibited cell metastasis of BCa, which could be rescued by *circSTX6*. The discovery of *circSTX6*/*miR-515-3p*/*SUZ12* axis represents a promising step for therapeutic intervention against tumors.

Apart from functioning as miRNA sponge, growing evidence indicated that circRNAs could interact with RBPs to participate in the regulation of tumor-related genes. The patterns of circRNA-protein interactions include as follows: (i) protein scaffold, in which circRNAs bring different proteins into proximity [58]; (ii) protein recruiter, in which specific circRNAs may recruit proteins to specific subcellular compartments [59]; (iii) protein decoy, in which circRNAs compete for binding to specific RBPs with other molecules sharing RBP binding domains [60]; (IV) protein function enhancer, in which individual circRNAs bind to and promote the functions of its RBPs [61]. For instance, *circNDUFB2* functions as a scaffold to enhance the interaction between TRIM25 and IGF2BPs, which is enhanced by m^6^A modifications of *circNDUFB2* [35]. Another study identified that *circRHOT1* induces the expression of NR2F6 by recruiting TIP60 to the promoter region of *NR2F6*, thereby initiating *NR2F6* transcription [62]. Yang et al. found that *circPTK2* functions as a protein decoy by blocking the phosphorylation sites of the vimentin protein to protect it from phosphorylation, leading to upregulation of vimentin [63]. In addition, m^6^A-modified *circNSUN2* acts as an enhancer of protein function by forming a ternary complex with IGF2BP2 protein and the *HMGA2* mRNA, thus promoting the stability of *HMGA2* mRNA [61]. In this study, we analyzed the *circSTX6* interacting protein through the online database and RNA pulldown experiments. Following a series of experiments, we further confirmed that circSTX6 could bind the RRM1-2 domain of PABPC1 protein to increase its interaction with SUZ12, resulting in promotion of SUZ12 mRNA stability. Significantly, our study demonstrates that *circSTX6* drives BCa metastasis and CDDP resistance by increasing SUZ12 mRNA expression and stability in a ceRNA- and RBP-dependent manner, which extends our knowledge of circRNAs in regulation of mRNA through dual-faceted regulation pathway.

Increasing evidence demonstrated that RNA drugs, including mRNA, shRNA, siRNA and ASO, have been approved to enter the clinical trial stage or clinical practice [64, 65]. Our study found that shRNAs targeting *circSTX6* suppressed BCa metastasis and CDDP chemoresistance, which could serve as a feasible treatment strategy for BCa. However, safety and targetability of these agents remains a major challenge. Previous study has identified small molecular compounds 5 and 16 specifically targeting the lncRNA MALAT1 [66], suggesting that ncRNAs can be targets for small-molecule drugs. Thus, *circSTX6* may be a potential target for developing small-molecule drugs to overcome metastasis and cisplatin resistance, which could shed light on the individual management of BCa.

## Conclusion

In conclusion, our present study demonstrates that *circSTX6* is significantly upregulated in both BCa cells and clinical samples. We also find that elevated *circSTX6* could effectively promote BCa cell metastasis and CDDP resistance. Mechanistically, we first discover that *circSTX6* mediated by EIF4A3 functions as a sponge of *miR-515-3p*, and in turn promotes the expression of SUZ12. More importantly, *circSTX6* cooperates with PABPC1 protein to promote downstream gene SUZ12 expression via improving its mRNA stability. Our finds reveal that therapeutic targeting of the *circSTX6* is a potential strategy for CDDP-resistant bladder cancer.

### Supplementary Information

**Additional file 1: Table S1.** Clinical features of 16 BCa patients and the expression of *circSTX6*

**Additional file 2**

**Additional file 3.**

**Additional file 4.**

**Additional file 5: Fig S1.***CircSTX6 *promotes the metastasis in BCa. A The knockdown and efficiency of *circSTX6 *in UMUC3 cells was detected by qRT-PCR. B The overexpression efficiency of *circSTX6 *in UMUC3 cells was detected by qRT-PCR. C Representative and quantified results of the Transwell migration and invasion assays in UMUC3 cells transfected with scramble, sh-circ#1, sh-circ#2, vector or circSTX6. Scale bar, 100 μm. D Cell migratory capabilities were assessed by wound healing assays after knocking down or overexpressing *circSTX6 *in UMUC3 cells. Scale bar, 400 μm. E CCK-8 assays showed the viability of BCa cells stably transfected with vector or circSTX6. F Edu assays depicted the proliferation rate of BCa cells stably transfected with scramble, sh-circ#1 or sh-circ#2. Scale bar, 200 μm. The data are presented as the means ± S.D. of at least three independent experiments. *P < 0.05. *P *values are calculated by Student’s t test in B and C and one-way ANOVA in A and C. **Fig S2. ***MiR-515-3p *inhibits BCa migration and invasion *in vitro*. A-B The influence on cell migration and invasion abilities of EJ (A) and UMUC3 (B) cells transfected with miR-NC, miR-515-3p, anti-miR-NC or anti miR-515-3p. Scale bar, 100 μm. C-D Cell migratory capabilities were assessed in BCa cells transfected with miR-NC, miR-515-3p, anti-miR-NC or anti miR-515-3p by wound healing assays. Scale bar, 400 μm. E Wound healing assays showed the migration abilities in EJ cells transfected with scramble or sh-circ#1, and those co-transfected with anti-miR-NC or anti miR-515-3p. Scale bar, 400 μm. The data are presented as the means ± S.D. of at least three independent experiments. *P < 0.05. *P* values are calculated by Student’s t test in A-D and one-way ANOVA in E. **Fig S3.** *CircSTX6* enhances the SUZ12 expression in BCa. A The mRNA and protein level of SUZ12 in UMUC3 cells stably transfected scramble, sh-circ#1, sh-circ#2, vector or circSTX6. B The expression level of *SUZ12 *in the TCGA BLCA dataset. C Western blot were performed to evaluate the expression of SUZ12 in EJ and UMUC3 cells which were transfected with scramble or sh-circ#1, and those co-transfected with mock or SUZ12. The data are presented as the means ± S.D. of at least three independent experiments. *P < 0.05. *P* values are calculated by Student’s t test in A (right) and one-way ANOVA in A (left).**Fig S4.** Knockdown of SUZ12 inhibits BCa migration and invasion *in vitro*. A Transwell assays showed the migration and invasion of UMUC3 cells transfected with mock, SUZ12, scramble or sh-SUZ12#1. Scale bar, 100 μm. B-C Cell migratory capabilities were assessed in EJ and UMUC3 cells transfected with with mock, SUZ12, scramble or sh-SUZ12#1 by wound healing assays. Scale bar, 400 μm. The data are presented as the means ± S.D. of at least three independent experiments. *P < 0.05. *P*values are calculated by Student’s t test in A-C. **Fig S5.** PABPC1 could facilitate the expression level of SUZ12 in UMUC3 cells. A The efficiency of PABPC1 knockdown or overexpression in UMUC3 cells was verified by qRT-PCR (left) and western blot (right). B qRT-PCR assay showed the expression of *circSTX6 *in UMUC3 cells transfected with the indicated plasmids. C qRT-PCR and western blot assays showed the expression of SUZ12 in UMUC3 cells transfected with the indicated plasmids. The data are presented as the means ± S.D. of at least three independent experiments. *P < 0.05. *P *values are calculated by Student’s t test in A and C and one-way ANOVA in A and C. **Fig S6.** Silencing of *circSTX6*/SUZ12 complex promotes the chemosensitivity of BCa cells to CDDP. A Determination of IC50 values for CDDP treatment 24 h in EJ cells which were stably transfected with mock or SUZ12 vector. B Flow cytometry apoptosis assays were used to assess apoptosis rate of UMUC3 cells stably transfected with sh-SUZ12#1 after CDDP treatment. C Determination of IC50 values for CDDP treatment 24 h in EJ cells which were stably transfected with vector or circSTX6 plasmid. D Flow cytometry apoptosis assays were used to assess apoptosis rate of UMUC3 cells stably transfected with sh-circSTX6 after CDDP treatment. E Tumor sphere formation assays were used to assess the self-renewal capacity of BCa treated with CDDP. The scale bar indicates 100 μm. F-G Immunofluorescence staining showed the expression of γH2AX in BCa cells treated with CDDP. The scale bar indicates 20 μm. H-I Immunofluorescence staining showed the expression of γH2AX in BCa cells treated with CDDP. The scale bar indicates 20 μm. J qRT-PCR assay showed the relative expression levels of *circSTX6 *in the EJ and EJ-CDDP cell lines. K Determination of IC50 values for CDDP treatment 24 h in EJ-CDDP cells which were stably transfected with scramble, sh-circ#1 or sh-circ#2 vector. L-M Flow cytometry assay revealed the CDDP effect in EJ-CDDP cells transfected with scramble, sh-circ#1 or sh-circ#2. N Flow cytometry assay revealed the CDDP effect in EJ-CDDP cells transfected with vector or circSTX6, and those co-transfected with scramble or sh-SUZ12#1. The data are presented as the means ± S.D. of at least three independent experiments. *P < 0.05. *P *values are calculated by Student’s t test in B, D, G, I and J and one-way ANOVA in M and N.

## Data Availability

All data generated or analyzed during this study are included in this published article and its Additional Files.

## Abbreviations

BCa: Bladder cancer
CircRNAs: Circular RNAa
CDDP: Cisplatin
STX6: Syntaxin 6
SUZ12: Suppressor of zeste 12 protein homolog
EIF4A3: Eukaryotic translation initiation factor 4A3
RBP: RNA-binding protein
PABPC1: Poly(A) binding protein cytoplasmic 1
TCGA: The Cancer Genome Atlas
gDNA: genomic DNA
NATs: Normal adjacent tissues
FISH: Fluorescence in situ hybridization
RIP: RNA immunoprecipitation
IF: Immunofluorescence
RISC: RNA-induced silencing complex
GSEA: Gene Set Enrichment Analysis
RRMs: RNA recognition motifs
