# Supplementary material for: EIF4A3-mediated biogenesis of circSTX6 promotes bladder cancer metastasis and cisplatin resistance
Source: J Exp Clin Cancer Res. 2024 Jan 2;43:2. doi: 10.1186/s13046-023-02932-6 (PMC10759346; doi:10.1186/s13046-023-02932-6)
Supplement: Supplementary file 1 — Additional file 1: Table S1. Clinical features of 16 BCa patients and the expression of circSTX6 [file 13046_2023_2932_MOESM1_ESM.docx]

**Table S1. Clinical features of 16 BCa patients and the expression of *circSTX6*.**

| **Parameters** | **Group** | **Cases** | ***CircSTX6* expression**  **High Low** | ***p*-value** |
| --- | --- | --- | --- | --- |
| Gender  Age at surgery  Pathological stage  Grade  Muscle invasion  Lymph node metastasis  Total | Male  Female  <60  ≥60  pTa-T1  pT2-T4  Low  High  NMIBC  MIBC  Absent  Present | 13  3  3  13  7  9  6  10  7  9  12  4  16 | 7 6  1 2  3 0  5 8  1 6  7 2  0 6  8 2  0 7  8 1  5 7  3 1  8 8 | >0.9999  0.2000  0.0406  0.0070  0.0014  0.5692 |

*P* < 0.05 represents statistical significance (Chi-square or Fish’ exact test)
