# Supplementary material for: EIF4A3-mediated biogenesis of circSTX6 promotes bladder cancer metastasis and cisplatin resistance
Source: J Exp Clin Cancer Res. 2024 Jan 2;43:2. doi: 10.1186/s13046-023-02932-6 (PMC10759346; doi:10.1186/s13046-023-02932-6)
Supplement: Supplementary file 5 — Additional file 5: Fig S1.CircSTX6 promotes the metastasis in BCa. A The knockdown and efficiency of circSTX6 in UMUC3 cells was detected by qRT-PCR. B The overexpression efficiency of circSTX6 in UMUC3 cells was detected by qRT-PCR. C Representative and quantified results of the Transwell migration and invasion assays in UMUC3 cells transfected with scramble, sh-circ#1, sh-circ#2, vector or circSTX6. Scale bar, 100 μm. D Cell migratory capabilities were assessed by wound healing assays after knocking down or overexpressing circSTX6 in UMUC3 cells. Scale bar, 400 μm. E CCK-8 assays showed the viability of BCa cells stably transfected with vector or circSTX6. F Edu assays depicted the proliferation rate of BCa cells stably transfected with scramble, sh-circ#1 or sh-circ#2. Scale bar, 200 μm. The data are presented as the means ± S.D. of at least three independent experiments. *P < 0.05. P values are calculated by Student’s t test in B and C and one-way ANOVA in A and C. Fig S2. MiR-515-3p inhibits BCa migration and invasion in vitro. A-B The influence on cell migration and invasion abilities of EJ (A) and UMUC3 (B) cells transfected with miR-NC, miR-515-3p, anti-miR-NC or anti miR-515-3p. Scale bar, 100 μm. C-D Cell migratory capabilities were assessed in BCa cells transfected with miR-NC, miR-515-3p, anti-miR-NC or anti miR-515-3p by wound healing assays. Scale bar, 400 μm. E Wound healing assays showed the migration abilities in EJ cells transfected with scramble or sh-circ#1, and those co-transfected with anti-miR-NC or anti miR-515-3p. Scale bar, 400 μm. The data are presented as the means ± S.D. of at least three independent experiments. *P < 0.05. P values are calculated by Student’s t test in A-D and one-way ANOVA in E. Fig S3. CircSTX6 enhances the SUZ12 expression in BCa. A The mRNA and protein level of SUZ12 in UMUC3 cells stably transfected scramble, sh-circ#1, sh-circ#2, vector or circSTX6. B The expression level of SUZ12 in the TCGA BLCA dataset. C Weste [file 13046_2023_2932_MOESM5_ESM.pdf]

Fig.S1

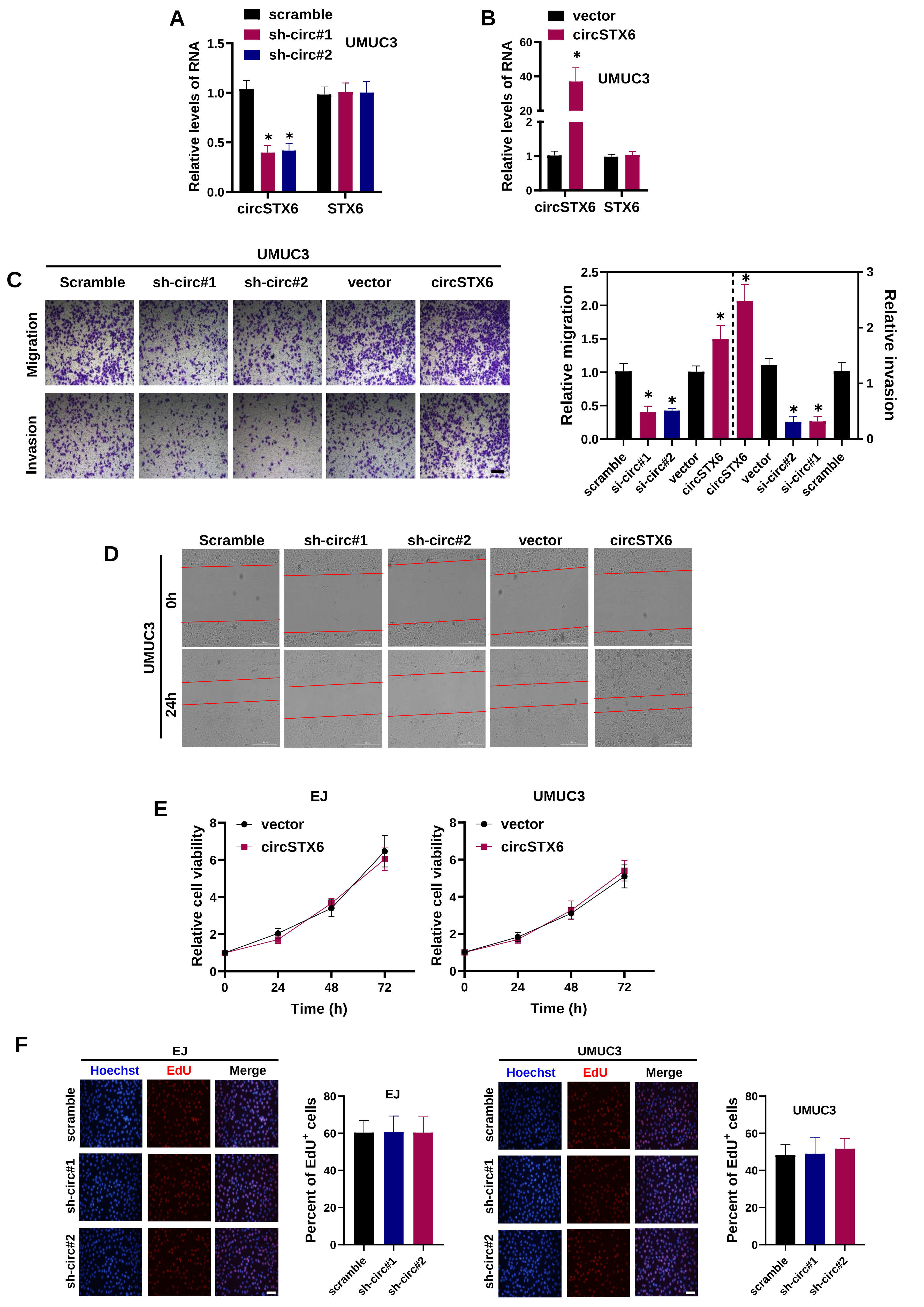
A
scramble
sh-circ#1
sh-circ#2
UMUC3
Relative levels of RNA
circSTX6
STX6
B
vector
circSTX6
UMUC3
Relative levels of RNA
circSTX6
STX6
C
UMUC3
Scramble
sh-circ#1
sh-circ#2
vector
circSTX6
Migration
Invasion
Relative migration
Relative invasion
scramble
si-circ#1
si-circ#2
vector
circSTX6
circSTX6
vector
si-circ#2
si-circ#1
scramble
D
Scramble
sh-circ#1
sh-circ#2
vector
circSTX6
UMUC3
0h
24h
E
EJ
UMUC3
vector
circSTX6
Relative cell viability
Time (h)
F
EJ
Hoechst
EdU
Merge
scramble
sh-circ#1
sh-circ#2
Percent of EdU+ cells
UMUC3

FigS1. *CircSTX6* promotes the metastasis in BCa. **A** The knockdown and efficiency of *circSTX6* in UMUC3 cells was detected by qRT-PCR. **B** The overexpression efficiency of *circSTX6* in UMUC3 cells was detected by qRT-PCR. **C** Representative and quantified results of the Transwell migration and invasion assays in UMUC3 cells transfected with scramble, sh-circ#1, sh-circ#2, vector or circSTX6. Scale bar, 100 μm. **D** Cell migratory capabilities were assessed by wound healing assays after knocking down or overexpressing *circSTX6* in UMUC3 cells. Scale bar, 400 μm. **E** CCK-8 assays showed the viability of BCa cells stably transfected with vector or circSTX6. **F** Edu assays depicted the proliferation rate of BCa cells stably transfected with scramble, sh-circ#1 or sh-circ#2. Scale bar, 200 μm. The data are presented as the means ± S.D. of at least three independent experiments. *$P < 0.05$. P values are calculated by Student's t test in **B** and **C** and one-way ANOVA in **A** and **C**.

Fig.S2

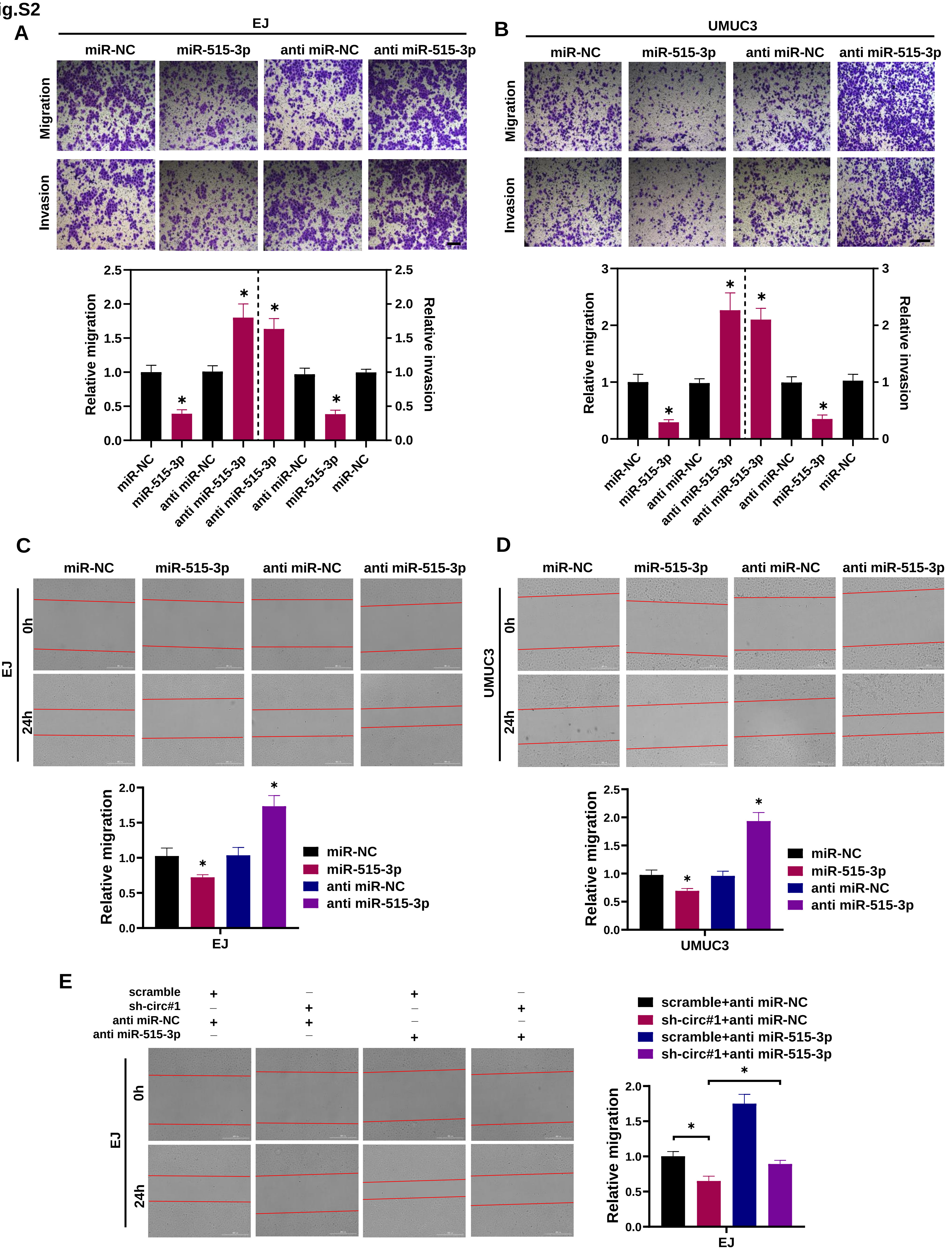
A
EJ
miR-NC
miR-515-3p
anti miR-NC
anti miR-515-3p
Migration
Invasion
Relative migration
Relative invasion
B
UMUC3
C
0h
24h
D
E
scramble
sh-circ#1
anti miR-NC
anti miR-515-3p
scramble+anti miR-NC
sh-circ#1+anti miR-NC
scramble+anti miR-515-3p
sh-circ#1+anti miR-515-3p

FigS2. *miR-515-3p* inhibits BCa migration and invasion *in vitro*. **A-B** The influence on cell migration and invasion abilities of EJ (A) and UMUC3 (B) cells transfected with miR-NC, miR-515-3p, anti-miR-NC or anti miR-515-3p. Scale bar, 100 μm. **C-D** Cell migratory capabilities were assessed in BCa cells transfected with miR-NC, miR-515-3p, anti-miR-NC or anti miR-515-3p by wound healing assays. Scale bar, 400 μm. **E** Wound healing assays showed the migration abilities in EJ cells transfected with scramble or sh-circ#1, and those co-transfected with anti-miR-NC or anti miR-515-3p. Scale bar, 400 μm. The data are presented as the means ± S.D. of at least three independent experiments. *P < 0.05. P values are calculated by Student's t test in **A-D** and one-way ANOVA in **E**.

Fig.S3

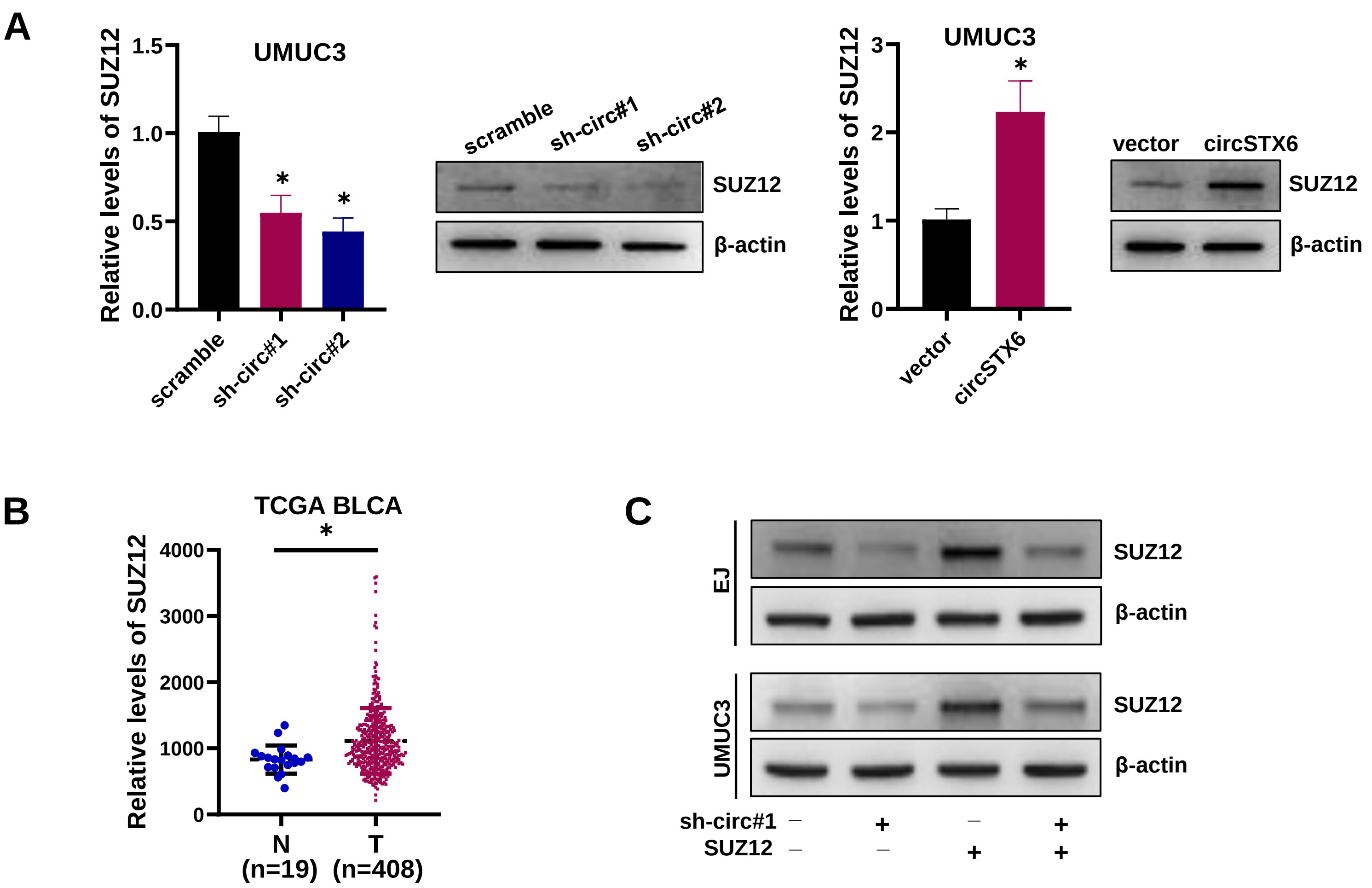

Fig S3. *CircSTX6* enhances the SUZ12 expression in BCa. **A** The mRNA and protein level of SUZ12 in UMUC3 cells stably transfected scramble, sh-circ#1, sh-circ#2, vector or circSTX6. **B** The expression level of *SUZ12* in the TCGA BLCA dataset. **C** Western blot were performed to evaluate the expression of SUZ12 in EJ and UMUC3 cells which were transfected with scramble or sh-circ#1, and those co-transfected with mock or SUZ12. The data are presented as the means ± S.D. of at least three independent experiments. *P < 0.05. P values are calculated by Student's t test in **A** (right) and one-way ANOVA in **A** (left).

Fig.S4

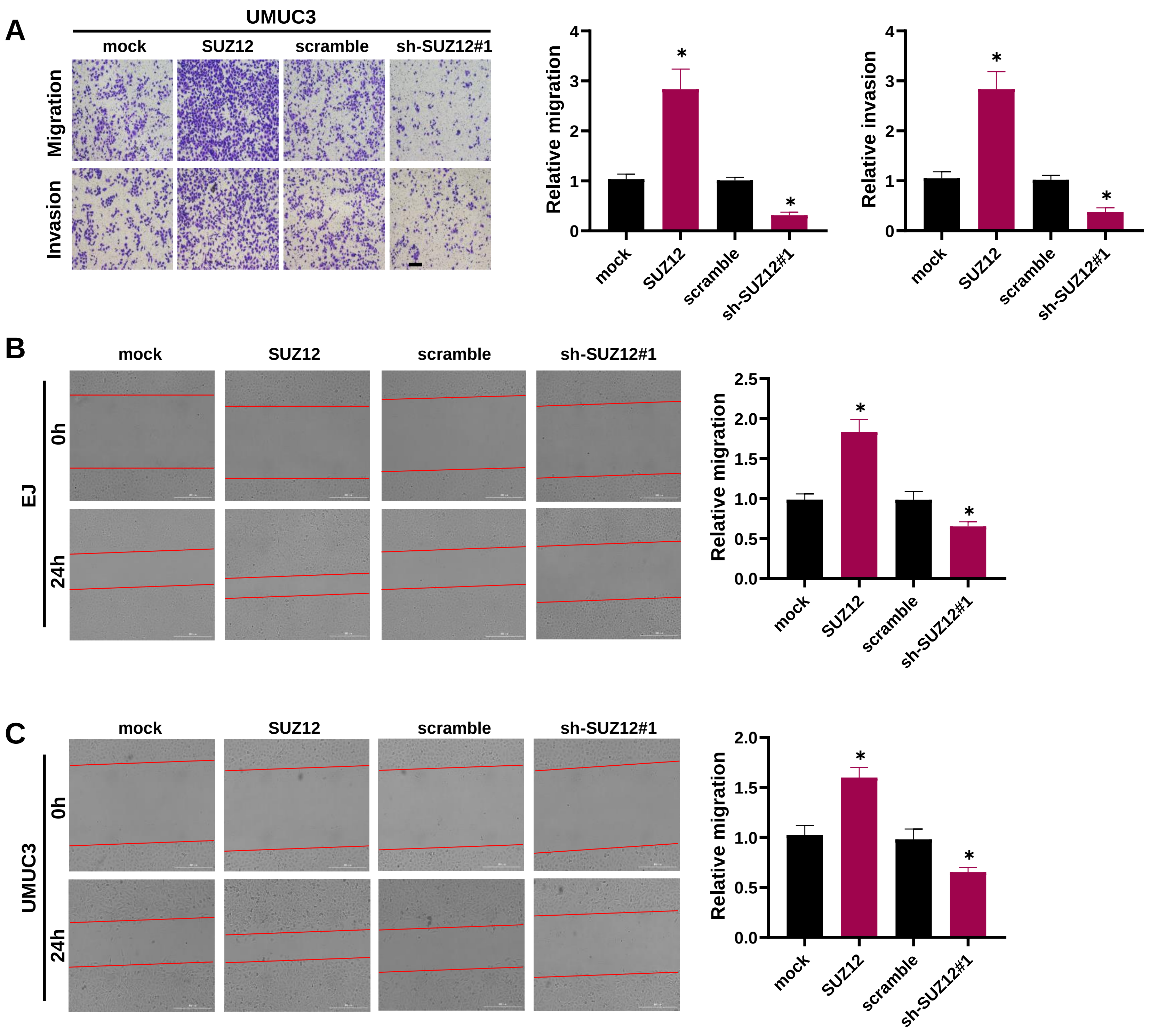

FigS4. Knockdown of SUZ12 inhibits BCa migration and invasion *in vitro*. **A** Transwell assays showed the migration and invasion of UMUC3 cells transfected with mock, SUZ12, scramble or sh-SUZ12#1. Scale bar, 100 μm. **B-C** Cell migratory capabilities were assessed in EJ and UMUC3 cells transfected with with mock, SUZ12, scramble or sh-SUZ12#1 by wound healing assays. Scale bar, 400 μm. The data are presented as the means ± S.D. of at least three independent experiments. **P* < 0.05. *P* values are calculated by Student's t test in **A-C**.

Fig.S5

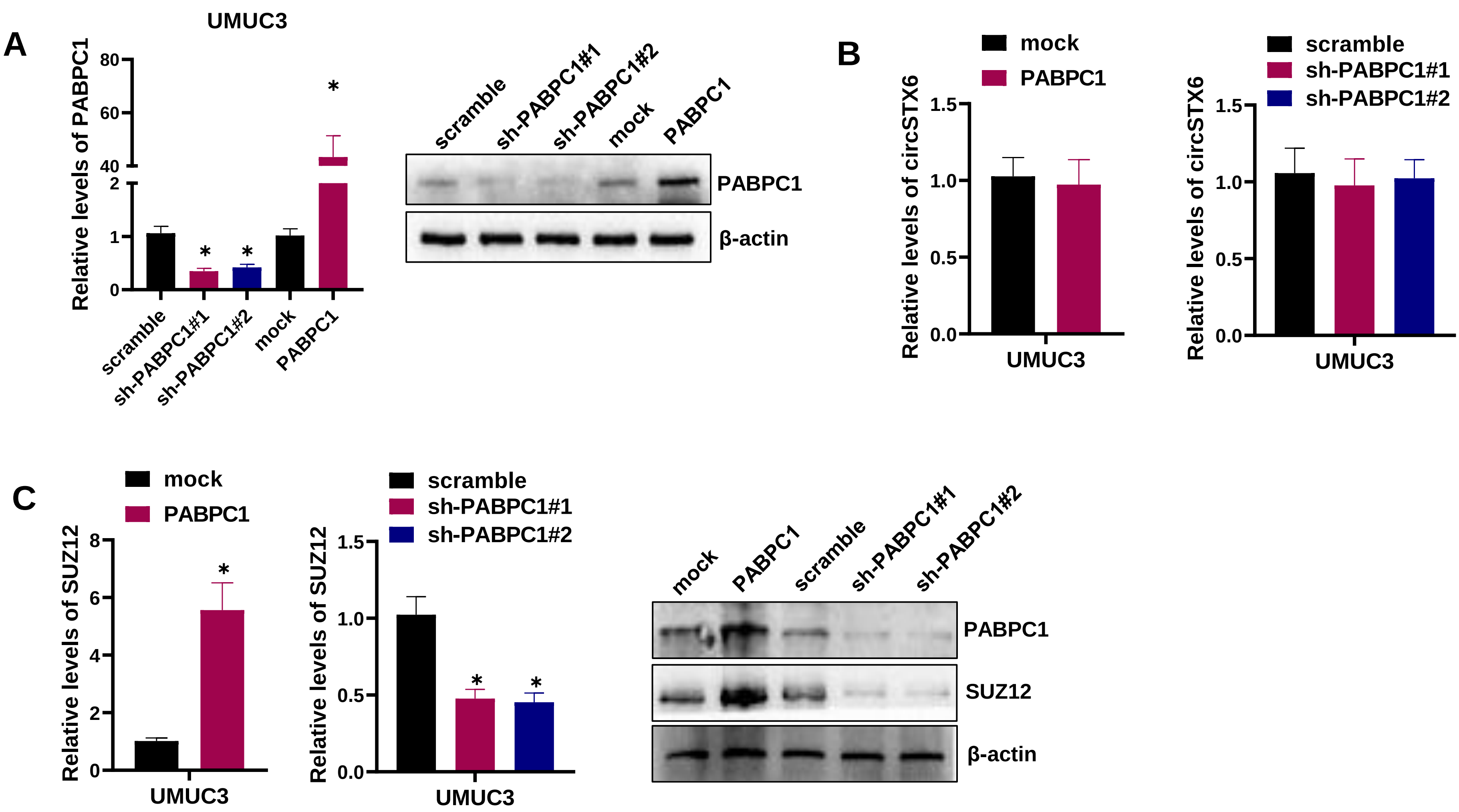

Fig S5. PABPC1 could facilitate the expression level of SUZ12 in UMUC3 cells. **A** The efficiency of PABPC1 knockdown or overexpression in UMUC3 cells was verified by qRT-PCR (left) and western blot (right). **B** qRT-PCR assay showed the expression of *circSTX6* in UMUC3 cells transfected with the indicated plasmids. **C** qRT-PCR and western blot assays showed the expression of SUZ12 in UMUC3 cells transfected with the indicated plasmids. The data are presented as the means ± S.D. of at least three independent experiments. *P < 0.05. P values are calculated by Student's t test in **A** and **C** and one-way ANOVA in **A** and **C**.

Fig.S6

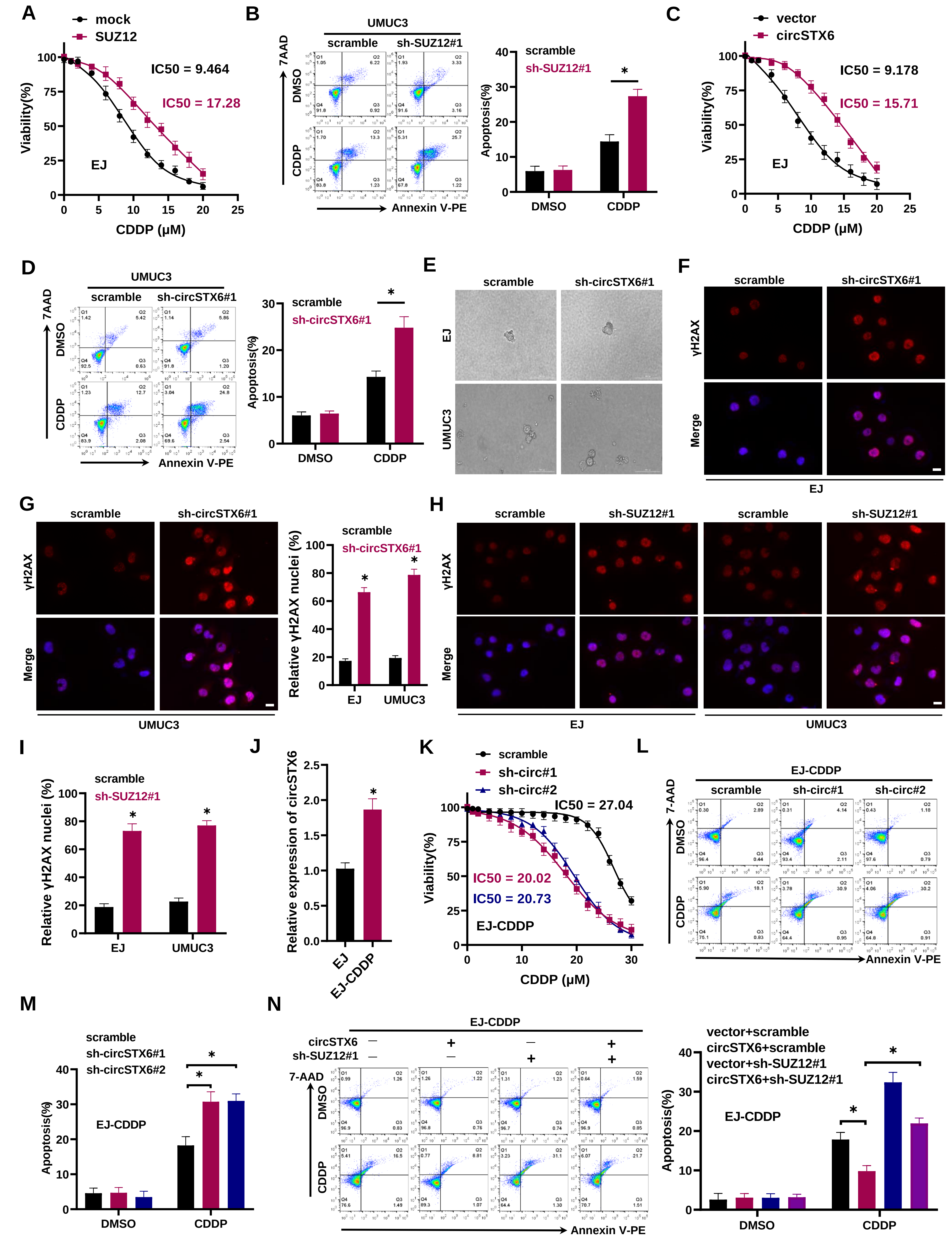
A
mock
SUZ12
IC50 = 9.464
IC50 = 17.28
EJ
Viability(%)
CDDP (μM)
B
UMUC3
scramble
sh-SUZ12#1
7AAD
DMSO
CDDP
Annexin V-PE
Apoptosis(%)
scramble
sh-SUZ12#1
DMSO
CDDP
C
vector
circSTX6
IC50 = 9.178
IC50 = 15.71
EJ
Viability(%)
CDDP (μM)
D
UMUC3
scramble
sh-circSTX6#1
7AAD
DMSO
CDDP
Annexin V-PE
Apoptosis(%)
scramble
sh-circSTX6#1
DMSO
CDDP
E
scramble
sh-circSTX6#1
EJ
UMUC3
F
scramble
sh-circSTX6#1
γH2AX
Merge
EJ
G
scramble
sh-circSTX6#1
γH2AX
Merge
UMUC3
Relative γH2AX nuclei (%)
scramble
sh-circSTX6#1
EJ
UMUC3
H
scramble
sh-SUZ12#1
scramble
sh-SUZ12#1
γH2AX
Merge
EJ
UMUC3
I
Relative γH2AX nuclei (%)
scramble
sh-SUZ12#1
EJ
UMUC3
J
Relative expression of circSTX6
EJ
EJ-CDDP
K
scramble
sh-circ#1
sh-circ#2
IC50 = 27.04
IC50 = 20.02
IC50 = 20.73
EJ-CDDP
Viability(%)
CDDP (μM)
L
EJ-CDDP
scramble
sh-circ#1
sh-circ#2
7-AAD
DMSO
CDDP
Annexin V-PE
M
scramble
sh-circSTX6#1
sh-circSTX6#2
EJ-CDDP
Apoptosis(%)
DMSO
CDDP
N
EJ-CDDP
circSTX6
sh-SUZ12#1
7-AAD
DMSO
CDDP
Annexin V-PE
vector+scramble
circSTX6+scramble
vector+sh-SUZ12#1
circSTX6+sh-SUZ12#1
EJ-CDDP
Apoptosis(%)
DMSO
CDDP

Fig S6. Silencing of *circSTX6*/SUZ12 complex promotes the chemosensitivity of BCa cells to CDDP. **A** Determination of IC50 values for CDDP treatment 24 h in EJ cells which were stably transfected with mock or SUZ12 vector. **B** Flow cytometry apoptosis assays were used to assess apoptosis rate of UMUC3 cells stably transfected with sh-SUZ12#1 after CDDP treatment. **C** Determination of IC50 values for CDDP treatment 24 h in EJ cells which were stably transfected with vector or circSTX6 plasmid. **D** Flow cytometry apoptosis assays were used to assess apoptosis rate of UMUC3 cells stably transfected with sh-circSTX6 after CDDP treatment. **E** Tumor sphere formation assays were used to assess the self-renewal capacity of BCa treated with CDDP. The scale bar indicates 100 μm. **F-G** Immunofluorescence staining showed the expression of γH2AX in BCa cells treated with CDDP. The scale bar indicates 20 μm. **H-I** Immunofluorescence staining showed the expression of γH2AX in BCa cells treated with CDDP. The scale bar indicates 20 μm. **J** qRT-PCR assay showed the relative expression levels of *circSTX6* in the EJ and EJ-CDDP cell lines. **K** Determination of IC50 values for CDDP treatment 24 h in EJ-CDDP cells which were stably transfected with scramble, sh-circ#1 or sh-circ#2 vector. **L-M** Flow cytometry assay revealed the CDDP effect in EJ-CDDP cells transfected with scramble, sh-circ#1 or sh-circ#2. **N** Flow cytometry assay revealed the CDDP effect in EJ-CDDP cells transfected with vector or circSTX6, and those co-transfected with scramble or sh-SUZ12#1. The data are presented as the means ± S.D. of at least three independent experiments. *$P < 0.05$. P values are calculated by Student's t test in **B, D, G, I** and **J** and one-way ANOVA in **M** and **N**.
